# High-Mobility and Bias-Stable Field-Effect Transistors Based on Lead-Free Formamidinium Tin Iodide Perovskites

**DOI:** 10.1021/acsenergylett.3c01400

**Published:** 2023-10-02

**Authors:** Zhiwen Zhou, Qihua Li, Mojun Chen, Xuerong Zheng, Xiao Wu, Xinhui Lu, Shuxia Tao, Ni Zhao

**Affiliations:** † Department of Electronic Engineering, The Chinese University of Hong Kong, Shatin 999077, Hong Kong SAR, China; ‡ Materials Simulation & Modelling, Department of Applied Physics, Eindhoven University of Technology, 5600 MB Eindhoven, The Netherlands; § Smart Manufacturing Thrust, Systems Hub, The Hong Kong University of Science and Technology, Guangzhou 511458, China; ∥ Department of Physics, The Chinese University of Hong Kong, Shatin 999077, Hong Kong SAR, China

## Abstract

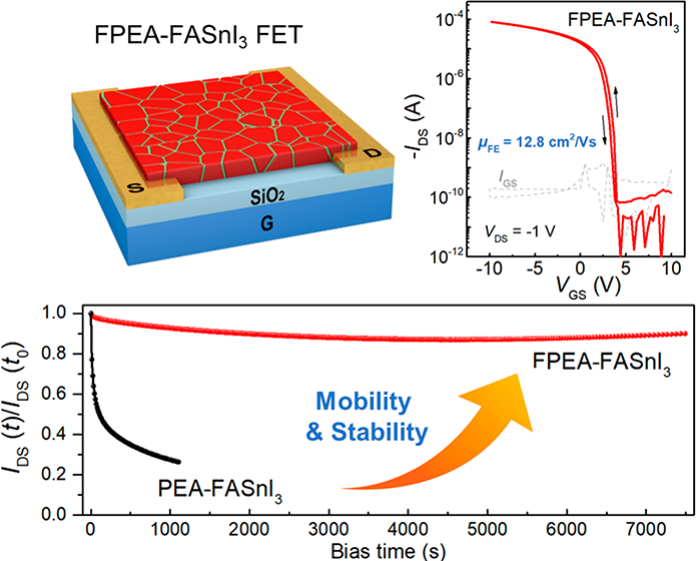

Electronic devices based on tin halide perovskites often exhibit a poor operational stability. Here, we report an additive engineering strategy to realize high-performance and stable field-effect transistors (FETs) based on 3D formamidinium tin iodide (FASnI_3_) films. By comparatively studying the modification effects of two additives, i.e., phenethylammonium iodide and 4-fluorophenylethylammonium iodide via combined experimental and theoretical investigations, we unambiguously point out the general effects of phenethylammonium (PEA) and its fluorinated derivative (FPEA) in enhancing crystallization of FASnI_3_ films and the unique role of fluorination in reducing structural defects, suppressing oxidation of Sn^2+^ and blocking oxygen and water involved defect reactions. The optimized FPEA-modified FASnI_3_ FETs reach a record high field-effect mobility of 15.1 cm^2^/(V·s) while showing negligible hysteresis. The devices exhibit less than 10% and 3% current variation during over 2 h continuous bias stressing and 4200-cycle switching test, respectively, representing the best stability achieved so far for all Sn-based FETs.

Tin halide perovskites (THPs) are one of the most promising candidates for realizing lead-free perovskite optoelectronic devices such as photovoltaic cells and transistors because they offer comparable optical and electronic properties as their Pb-halide analogues while offering lower toxicity.^1,2^ Moreover, THPs are expected to possess higher hole mobility and negligible ion migration because of weaker Fröhlich interactions and stronger Sn-halide bonds, which raises the ion migration activation energies in THPs.^3,4^ The first p-type THP-based field-effect transistor (FET) was demonstrated more than two decades ago, when a field-effect hole mobility (μ_FET_) of 0.6 cm^2^/(V·s) was reported for a two-dimensional (2D) phenethylammonium tin iodide (PEA_2_SnI_4_) system.^5^ Since then, attempts to improve the device performance and stability of 2D THP-based FETs have been carried out,^6−10^ but the improvement was rather limited, due possibly to the inherently poor charge transport properties of the perovskite films caused by the insulating organic spacers and strong quantum confinement effects.^11^ In addition, large current–voltage hysteresis has often been observed in these 2D THP-based FETs, which may be ascribed to the high density of traps.^12^

Unlike the 2D layer perovskites, three-dimensional (3D) tin perovskites feature a continuous 3D network of corner-shearing inorganic octahedrons that is free of charge-blocking organic spacers, thereby facilitating much faster charge transport in thin films.^13^ High carrier mobility of over 50 cm^2^/(V·s) has been recently demonstrated using a 3D Sn–Pb mixed perovskite system.^14,15^ Remarkably, the corresponding perovskite FETs also exhibited a very small hysteresis. Sirringhaus et al. have comprehensively investigated the charge transport physics of these 3D mixed Sn–Pb perovskite semiconductors, unveiling the significantly suppressed ionic migration effects and reduced hole effective mass by replacing Pb with Sn in the perovskite lattice.^16^ However, pure Sn-perovskite FETs still exhibit poor operational stability due to easy oxidation of Sn^2+^ to Sn^4+^ and high density of defects, such as tin vacancies (V_Sn_). Device degradation is often found to occur even in a nitrogen-filled glovebox with a trace amount of oxygen. To address the stability issue, hybrid 2D/3D THP systems were explored as the FET active layer. In the early attempts, the devices exhibited either limited carrier mobility (less than 1 cm^2^/(V·s))^13^ or substantial device hysteresis,^17^ due possibly to the poor film morphology or formation of defect-rich phases. During the preparation of this manuscript, Noh et al. reported the combinational use of antisolvent processing and fluorinated organic cation in achieving high-mobility (12 cm^2^/(V·s)) and hysteresis-free 2D/3D hybrid THP FETs.^18^ The study demonstrates the great impact of material and process optimization on the device performances and calls for fundamental understanding of the structure–property–stability correlations in pure THP systems.

In this work, we develop an additive engineering strategy to realize high-performance and stable 3D formamidinium tin iodide (FASnI_3_) based FETs. By employing a small amount of organic salt additive (i.e., phenethylammonium iodide (PEAI) or 4-fluorophenylethylammonium iodide (FPEAI)) in the perovskite precursor, highly crystalline FASnI_3_ films with pronounced preferential crystal orientation are obtained. A comprehensive comparative study combining ex situ structural and chemical analysis, in situ optical and electrical characterizations, and density functional theory (DFT) calculations reveals that the fluorinated phenethylammonium passivation is much more effective in reducing structural defects, suppressing oxidation of Sn^2+^ and blocking oxygen and water involved defect reactions. As a result, the optimized FET devices based on FPEA-modified FASnI_3_ show a high field-effect mobility of 15.1 cm^2^/(V·s), on/off current ratio over 10^7^, and negligible hysteresis. Most importantly, the devices exhibit excellent operational stability, with less than 10% and 3% current variation during over 2 h continuous bias stressing and 4200-cycle switching test, respectively.

## FET Performance

**Figure 1:**
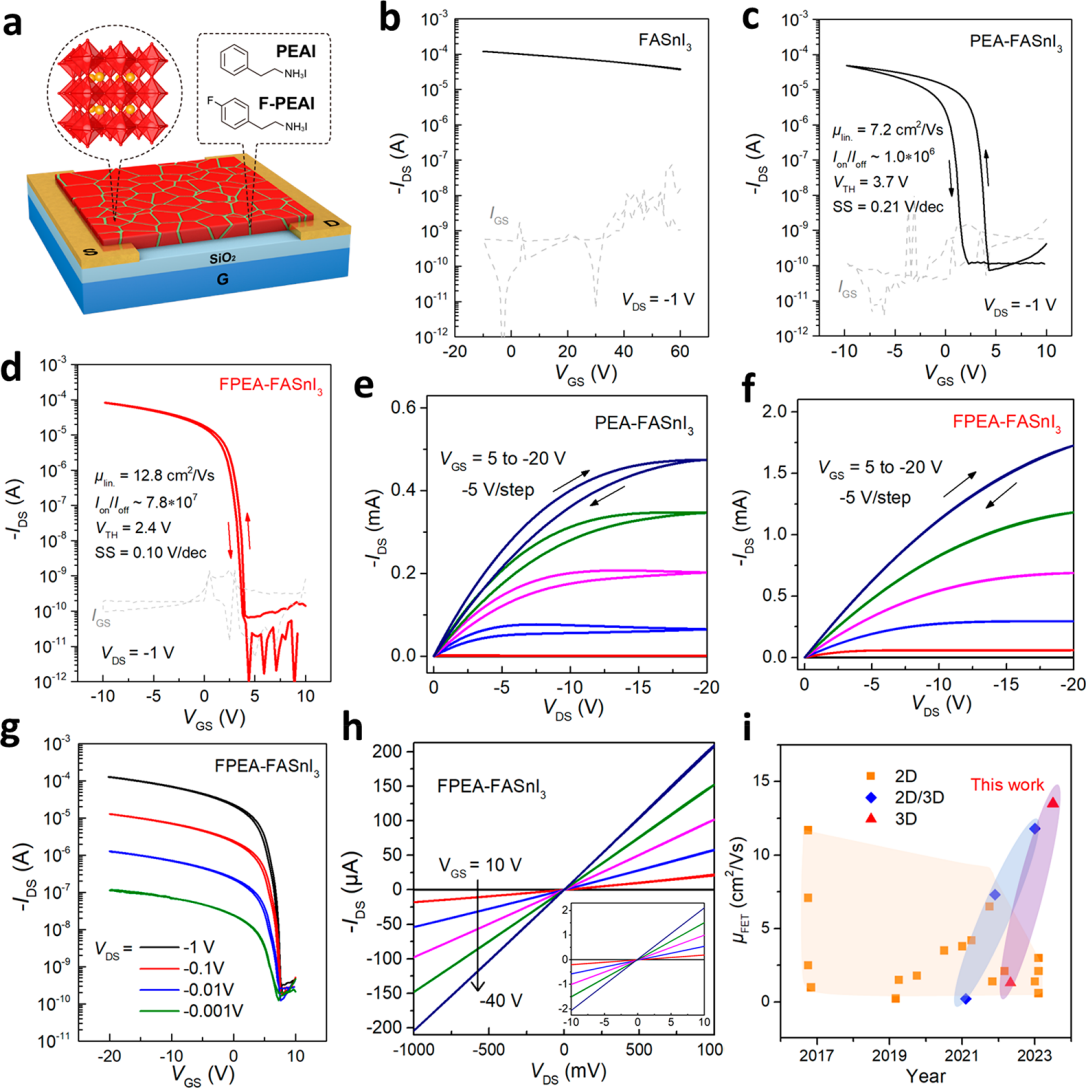
**Device characteristics of perovskite FETs**. (a) Schematic of the bottom-gate/bottom-contact FET structure used in this study. Typical transfer curves of FETs based on (b) neat FASnI_3_, (c) PEA-FASnI_3_, and (d) FPEA-FASnI_3_ films. Output curves of (e) PEA-FASnI_3_ and (f) FPEA-FASnI_3_ devices. (g) Dual-sweep linear transfer curves of one device based on the FPEA-FASnI_3_ film at different *V*_DS_ from −1 V to −1 mV. (h) The corresponding output curves scanning at a low *V*_DS_ region from −1 to 1 V. The inset is the magnification of the output scanning from *V*_DS_ = 10 to −10 mV. (i) Summarized field-effect mobility (μ_FET_) evolution of lead-free Sn-based perovskite FETs in the past 7 years. More details on device performance are listed in Table S1 in the Supporting Information.

As illustrated in Figure 1a, a bottom-gate, bottom-contact (BGBC) device configuration with a channel length/width of 100/1500 μm is adopted for device fabrication. (See Experimental Methods in the Supporting Information for the detailed device fabrication procedure.) Note that a small amount of SnF_2_ (10 mol% with respect to the SnI_2_) was incorporated into the perovskite precursors, which acts as a reducing agent to alleviate Sn^2+^ oxidation and reduce tin vacancies.^19,20^ We first compare the device performance of FETs with and without additive modification. As shown in Figure 1b, the unmodified FASnI_3_ FET shows very weak gate modulation due to the strong p-type self-doping effect. This result is consistent with previous reports on the same material system and can be explained by the prevalent formation of Sn vacancies due to the easy oxidation of Sn^2+^ to Sn^4+^ and the low defect formation energy in 3D pure tin perovskites.^16^ This also suggests that the addition of SnF_2_ alone is not sufficient to inhibit Sn^2+^ oxidation and reduce the background hole density. In contrast, the transistors based on the SnF_2_ and PEAI co-modified FASnI_3_ films (denoted by “PEA-FASnI_3_”) exhibit standard p-channel transfer curves, as shown in Figure 1c, with a field-effect mobility (μ_FET_) of 7.2 cm^2^/(V·s), an *I*_on_/*I*_off_ ratio over 10^6^, and a low subthreshold swing of 0.21 V/dec. However, a relatively large hysteresis (Δ*V*_H_ ≈ 2.6 V) is still observed from consecutive forward and reverse scans of the transistor (noted that the Δ*V*_H_ is defined as the gate voltage difference at the absolute *I*_DS_ value of 10^–7^ A, halfway between the on and off states^15^).

Surprisingly, replacing the PEAI with FPEAI significantly improves the transistor performance, resulting in negligible device hysteresis and improved mobility. As shown in Figure 1d, the representative FPEA-FASnI_3_ FET exhibits a μ_FET_ of 12.8 cm^2^/(V·s), *I*_on_/*I*_off_ ratio exceeding 10^7^, and a small subthreshold swing of 0.1 V/dec. Furthermore, the output curves of the FPEA-FASnI_3_ device exhibit ideal linear (ohmic) and saturation characteristics with negligible hysteresis (Figure 1f). Most importantly, the FPEA-FASnI_3_ devices exhibit excellent reproducibility in electrical performance, with an average linear μ_FET_ of 12.1 ± 0.5 cm^2^/(V·s) for 20 FPEA-FASnI_3_ transistors obtained in several batches (Figure S1). It is also worth noting that the FPEA-FASnI_3_ FETs can maintain excellent gate-induced current modulation even at an extremely small drain–source voltage (*V*_DS_) of −0.001 V (as shown in Figure 1g) and that highly linear and symmetric output characteristics are well preserved from 1 V to 10 mV (Figure 1h). Such a good linear property at low *V*_DS_ is highly desired for electronic applications such as signal processing sensors and active-matrix backplanes for LCD (liquid crystal display) and LED (light-emitting diode) displays. The optimized FPEA-FASnI_3_ FETs exhibit champion linear and saturated field-effect mobility values of 13.9 and 15.1 cm^2^/(V·s), respectively (Figures S2 and S3), representing the best performances achieved so far for lead-free 3D perovskite transistors (Figure 1i and Table S1).

## Device Stability Characterizations

**Figure 2:**
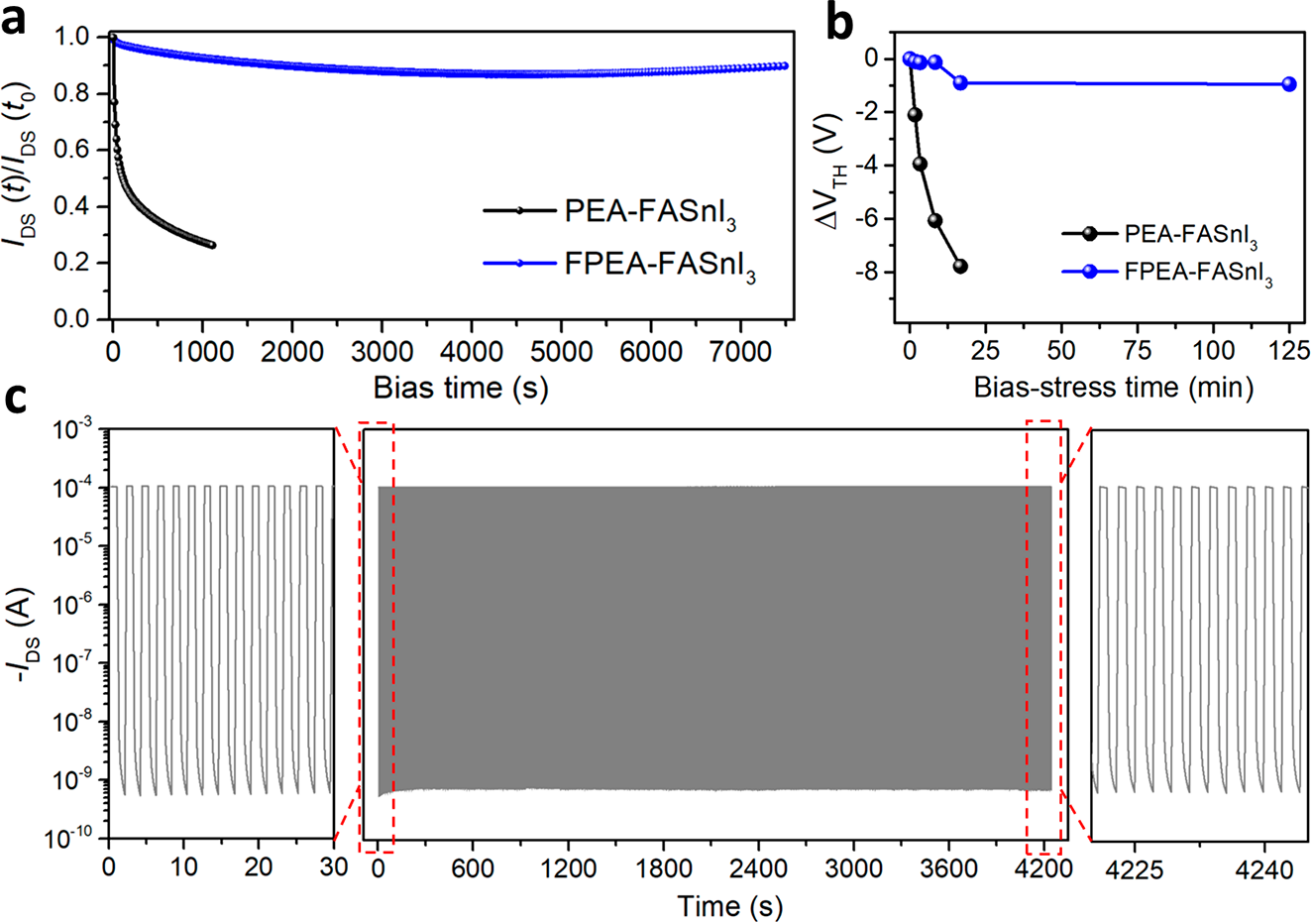
**Operational stability of perovskite FETs**. (a) Bias-stress stabilities of typical FETs based on PEA-FASnI_3_ and FPEA-FASnI_3_ films. A constant bias condition of *V*_GS_ = −10 V and *V*_DS_ = −1 V was used during the bias-stress measurements. (b) Corresponding variations of *V*_TH_ of PEA-FASnI_3_ and FPEA-FASnI_3_ devices under constant bias stress. (c) Continuous on/off switching test of one representative FPEA-FASnI_3_ transistor device.

To evaluate the operational stability of the perovskite FETs, we performed a prolonged bias-stress measurement under a constant negative gate and drain voltage (*V*_GS_ = −10 V, *V*_DS_ = −1 V) in a N_2_-filled glovebox. Remarkably, the FPEA-FASnI_3_ transistor exhibits excellent operational stability, with a *V*_TH_ variation of less than 1 V and *I*_DS_ decay of less than 10% after continuous biasing for more than 7500 s, i.e., more than 2 h, (Figure 2a,b), representing the most stable performances demonstrated in a 3D Sn-based perovskite transistor. In contrast, the PEA-FASnI_3_ device exhibits a fast decay of the *I*_DS_, accompanied by a large negative *V*_TH_ shift (∼8 V) after only 1000 s of bias-stress test. We note that the degree of the *I*_DS_ decay appears to be different in the PEA-FASnI_3_ devices fabricated from different batches (Figure S4a), suggesting that the degradation mechanism may be very sensitive to the fabrication or testing environment. Furthermore, the *I*_DS_ current after removing the bias failed to recover toward its initial state (Figure S4b), indicating the prolonged voltage bias might cause permanent material degradations.^21^ The underlying mechanism for such radically different bias stress stabilities will be explored in the later sections. The transfer characteristics of the above two representative devices at different bias durations are accordingly given in Figure S5.

The operational stability of the FPEA-FASnI_3_ FETs was also characterized by a dynamic on/off switching test. As shown in Figure 2c, the device provides highly reproducible on- and off-current states for more than 4200 cycles. Apart from the operational stability, we also examined the ambient stability and shelf life stability of the PEA-FASnI_3_ and FPEA-FASnI_3_ FETs. Without encapsulation, the PEA-FASnI_3_ device lost the transistor characteristics after 24 h exposure in air, while the FPEA-FASnI_3_ maintains the high mobility and high on/off ratio of the transistor, with only a few volts of *V*_TH_ shift (Figure S6). After encapsulation with a polyisobutylene (PIB) layer, the PEA-FASnI_3_ device shows much improved stability but still displays a large *V*_TH_ shift after 10 days of storage in a glovebox with an oxygen level of ∼25 ppm; in contrast, the *V*_TH_ variation of the FPEA-FASnI_3_ device is only about 1 V (Figure S7).

## Perovskite Film Characterizations

**Figure 3:**
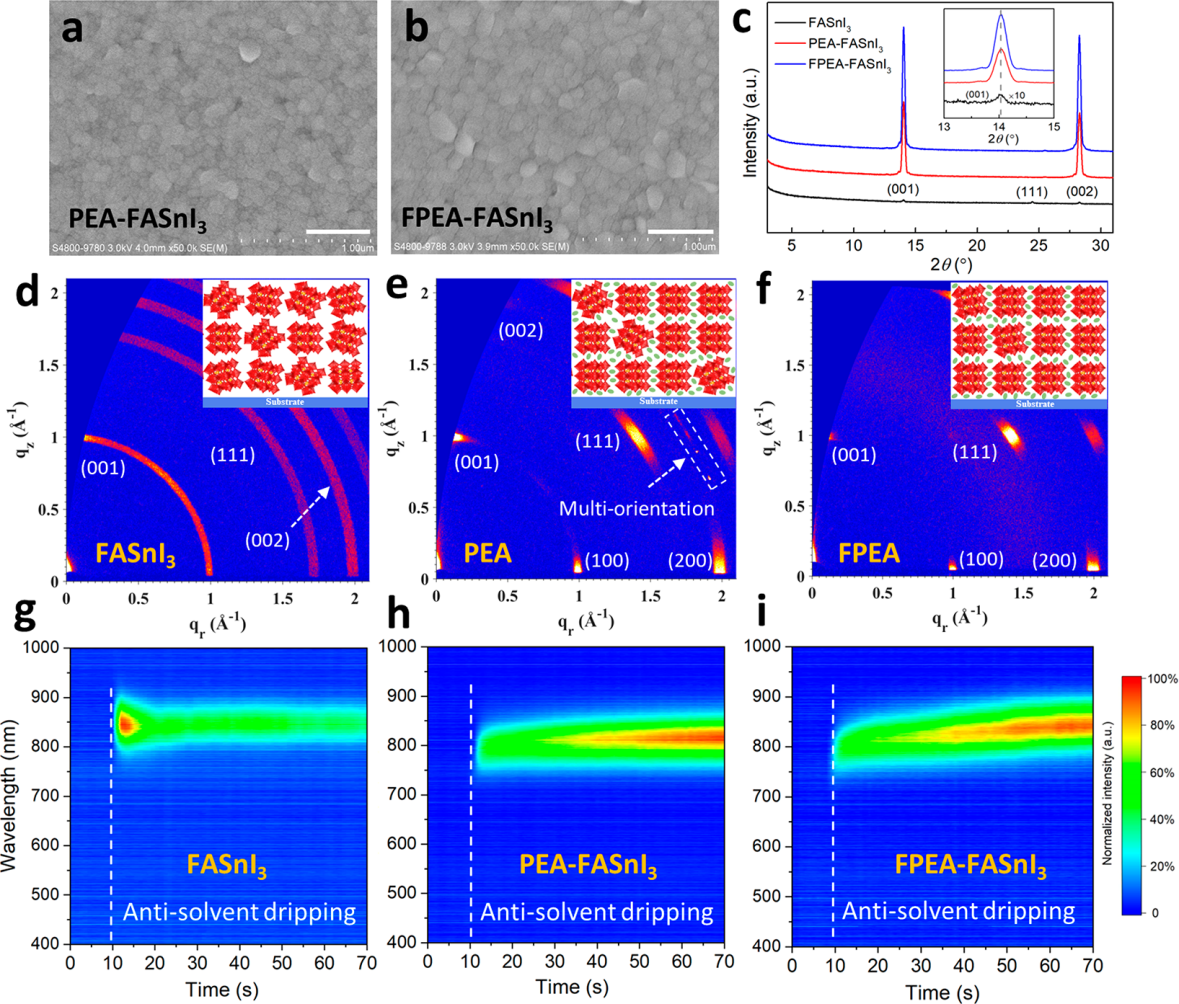
**Characterizations of perovskite films**. Typical SEM images of (a) PEA-FASnI_3_ films and (b) FPEA-FASnI_3_ films. (c) General θ – 2θ XRD patterns of pure FASnI_3_, PEA-FASnI_3_, and FPEA-FASnI_3_ films. GIWAXS images of (d) FASnI_3_, (e) PEA-FASnI_3_ and (f) FPEA-FASnI_3_ films. Inset figures in the top corner correspond to the schematic illustration of terminal crystal orientations of perovskite films. The green elliptical dots in the insets of (d, e) schematically represent the PEAI/FPEAI molecules. Note that these insets illustrate only the crystal orientation without implying the crystal size information. In situ photoluminescence (PL) mapping images of (g) FASnI_3_, (h) PEA-FASnI_3_, and (i) FPEA-FASnI_3_ films with a 10th antisolvent dripping process.

To understand the underlying mechanisms of the performance and stability improvements of the PEAI- or FPEAI-treated FASnI_3_ FETs, we first carried out morphological and structural characterization measurements on the perovskite films with and without PEAI/FPEAI modification. As shown in Figure S8, the pristine FASnI_3_ film exhibits poor surface coverage with a large number of voids throughout the film, which are associated with the rapid crystal growth process and the low solute concentration that was chosen to limit the active layer thickness to tens of nanometers for optimized transistor operation.^14^ In striking contrast, dense and pinhole-free thin films were obtained for both the PEA-FASnI_3_ and FPEA-FASnI_3_ cases (Figures 3a,b), indicating that the PEAI and FPEAI molecules play an important role in the film formation process. Furthermore, we found that the film morphology can be tuned by the precursor concentration (Figure S9) and accordingly optimized the precursor concentration to be 0.2 M. The crystallinity of the films also varies with the concentration of the PEAI/FPEAI additive (Figure S10), peaking at the same optimal additive concentration as the FET mobility (Figure S3). Notably, the FPEA-FASnI_3_ film has a more uniform grain distribution (grain size ranging from 100 to 200 nm) and larger grain size than the PEA-FASnI_3_ film (grain size ranging from 20 to 150 nm). Previous studies have shown that the grain boundaries (GBs) of perovskite films can accommodate a large number of structural defects, which serve as trap states to hinder charge transport,^6^ as well as providing pathways for infiltration of water and oxygen, thereby reducing the chemical stability of the devices.^22^ Therefore, the reduced GBs and increased grain size of the FPEA-FASnI_3_ films may also contribute to the superior electronic properties and device stability of the corresponding FET devices. In the following section, the crystallinity and crystallization process of the films are further studied.

X-ray diffraction (XRD) and grazing-incidence wide-angle X-ray scattering (GIWAXS) measurements were performed to evaluate the crystallinity and crystal orientation of the FASnI_3_ perovskite thin films. As shown in Figure 3c,d, the XRD and GIWAXS patterns of the pristine FASnI_3_ film indicate poor crystallinity and random crystal orientation, as evidenced by the presence of the weak peaks assigned to the (001), (111), and (002) crystal planes of a cubic phase.^23^ On the other hand, the PEAI- or FPEAI-treated FASnI_3_ films exhibit a drastically improved crystallinity and preferred orientation, as indicated by (1) the greatly enhanced (00*l*) peaks and nearly invisible (111) peak in the XRD patterns and (2) the bright Bragg spots or short arcs of the corresponding diffraction planes in the GIWAXS patterns (Figure 3e,f). The insets in Figure 3e,f illustrate the preferential orientation of the (00*l*) planes parallel to the substrate surface. Notably, no additional peaks are observed at low diffraction angles (or low *q* values), indicating the absence of 2D phase perovskites (or if any, a negligible amount of 2D perovskites) in the PEAI- and FPEAI-treated FASnI_3_ films. This absence of 2D perovskite phases differs from some previous reports where a large ratio (over 20 mol%) of ammonium ligands^18,24^ and a different solvent composition^25^ were employed in the precursor solutions to induce distinctive 2D/3D hybrid structures; on the other hand, the result is consistent with the studies where a similar amount of PEAI was used for fabricating 3D FASnI_3_ light-emitting diodes.^26,27^ Furthermore, the pristine and treated perovskite films show exactly the same diffraction peak positions (inset of Figure 3c), suggesting that the PEA and FPEA cations do not enter the 3D crystal lattice of FASnI_3_, consistent with previous reports.^21^ It is worth noting that the FPEA-FASnI_3_ perovskite film exhibits more intense (00*l*) diffraction peaks (Figure 3c) and narrower azimuthal angle of the (111) diffraction than the PEA-FASnI_3_ film (Figure S11), suggesting higher crystallinity and a stronger preferential orientation of the former.

To further understand how PEAI or FPEAI additives influence the crystal growth of the FASnI_3_ perovskite, we employed in situ PL measurement to dynamically monitor the film formation process. For the pristine FASnI_3_ film (Figure 3g), a strong PL peak corresponding to the perovskite phase appeared rapidly upon antisolvent dripping, indicating a very fast crystallization process. (The PL peak disappeared later on due to the poor stability of the neat FASnI_3_ perovskite under laser illustration.) In contrast, introducing PEAI or FPEAI molecules in the precursor solution significantly delays the appearance of the intense PL peak (Figure 3h,i), suggesting that the crystallization process of the perovskite has been greatly slowed down. According to previous studies, such retarded crystal growth is beneficial for the growth of perovskite grains with a preferential orientation^29,30^ and may explain the higher charge carrier mobility of the FPEA-FASnI_3_ FETs.

## Trap Analysis for Fresh Perovskite Films and Devices

**Figure 4:**
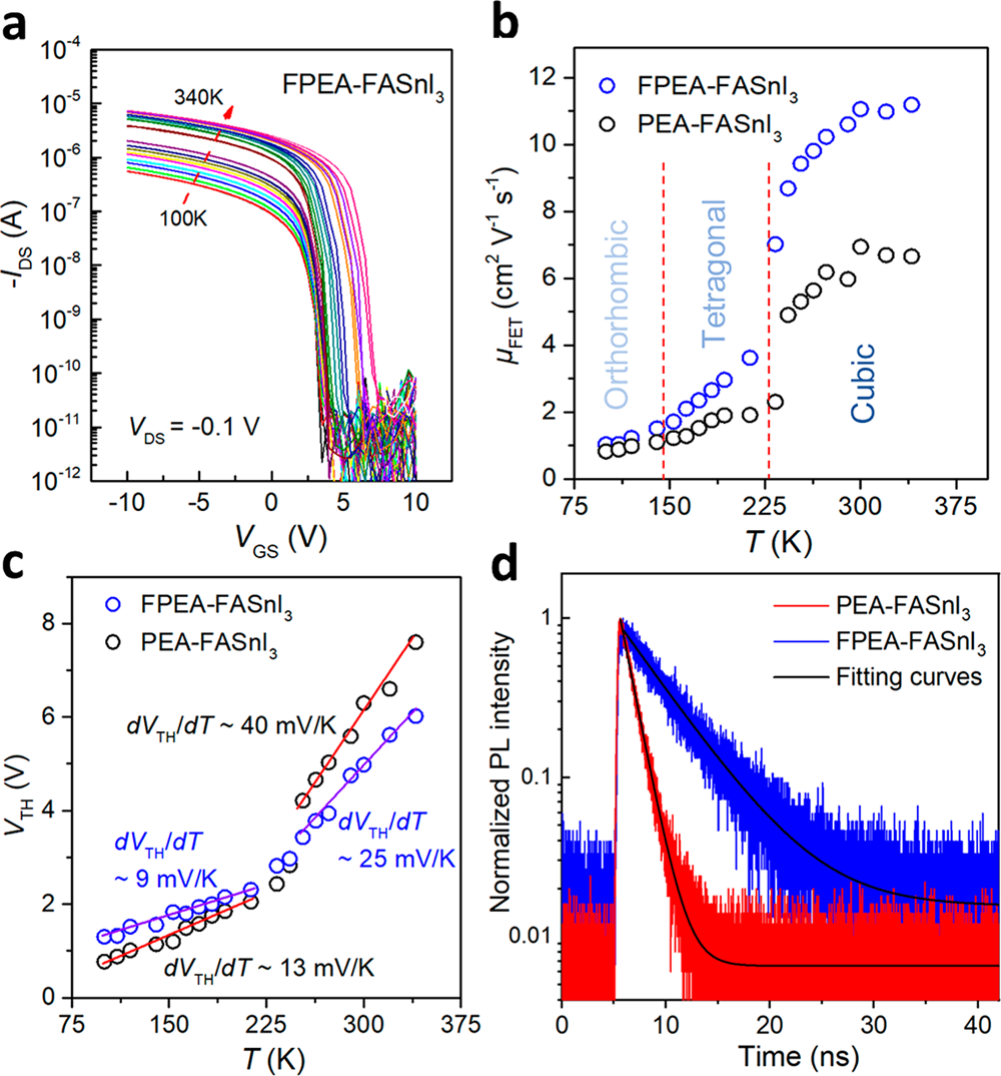
**Defect characterizations of perovskite films**. (a) Temperature-dependent electrical transfer curves measured on one typical FPEA-FASnI_3_ transistor under vacuum conditions. (b) Temperature-dependent field-effect hole mobilities for two typical FET devices based on PEA-FASnI_3_ and FPEA-FASnI_3_ films. The dotted lines roughly indicate the boundary of phase transitions of FASnI_3_ at different temperature ranges. (c) The corresponding temperature-dependent threshold voltages of these two typical FET devices. (d) Time-resolved PL spectroscopy (TRPL) decays of PEA-FASnI_3_ and FPEA-FASnI_3_ films.

Bias stress in FETs is often associated with the presence of intrinsic bulk or interface deep traps,^31,32^ electrical activated trap formation,^33,34^ or charge-induced electrochemical reactions.^35^ To characterize the intrinsic traps in the perovskite FETs, we performed temperature-dependent *I*–*V* measurements on freshly made devices. Here each transfer curve measurement was taken in only 20 s to minimize the device stress, and between each temperature point the device was rested for at least 15 min. Figure 4a presents the linear-regime transfer curves of a representative FPEA-FASnI_3_ device measured in a temperature range of 100–340 K. For comparison, the temperature-dependent transfer characteristics of a typical PEA-FASnI_3_ transistor are provided in the Supporting Information (Figure S12). The temperature-dependent hole mobilities calculated from the transfer curves of both devices are plotted in Figure 4b. It can be seen that in the 100–300 K range both the PEA-FASnI_3_ and FPEA-FASnI_3_ FETs exhibit a general trend of mobility increase with increasing temperature, indicating a thermally activated charge transport mechanism. Notably, a sudden drop of mobility was observed for both the transistors in the temperatures close to 225–250 K, which may be associated with the phase transition of FASnI_3_ from a cubic to tetragonal phase.^36^ In fact, below 300 K the temperature-dependent hole mobility characteristics can be divided into three distinct regions, corresponding to the cubic, tetragonal, and orthorhombic phases, respectively. In the following analysis, we only focus on the cubic phase (i.e., 250–300 K regime) as it is where we tested the operational stability of the devices.

By fitting the mobility data to an Arrhenius relation, μ = e^–*E*_a_/*kt*^, where *E*_a_ is the activation energy and *k* is Boltzmann’s constant, we extract the activation energies of ∼14.4 and ∼8.9 meV for the PEA-FASnI_3_ and FPEA-FASnI_3_ FETs, respectively. These results are comparable to the previously reported values for other Sn-based perovskite FETs,^10,16^ and suggest the dominance of shallow traps. Furthermore, the temperature-dependent threshold voltage (*V*_TH_) shift was plotted in Figure 4c. It can be seen that the *V*_TH_ vs *T* characteristic of the PEA-FASnI_3_ device exhibits a larger slope (∼40 mV/K) than that of the FPEA-FASnI_3_ device (∼25 mV/K). Using the equation *D_t_* = ,^37,38^ where *D*_t_ is the trap density, *C*_i_ represents the areal capacitance of the dielectric layer, and *e* is the elementary charge, we estimate the trap density to be ∼8.7 × 10^13^ cm^–2^ eV^–1^ for the PEA-FASnI_3_ device and ∼5.4 × 10^13^ cm^–2^ eV^–1^ for the FPEA-FASnI_3_ device.

We also performed steady-state and time-resolved PL spectroscopy on the perovskite films. Here, a low excitation power (10 μW) was used to better reveal the trap-assisted recombination process. Compared to PEA-FASnI_3_, the FPEA-FASnI_3_ system exhibits a higher PL intensity and longer carrier lifetime, as shown in Figure S13 and Figure 4d. Employing a monoexponential decay model, the carrier lifetimes are estimated to be 1.29 and 4.92 ns for PEA- FASnI_3_ and FPEA-FASnI_3_, respectively. The improved PL properties of the FPEA-FASnI_3_ films can be attributed to reduced number of recombination centers.^39^ Overall, both the electrical and spectroscopic measurement results suggest that the FPEA-FASnI_3_ system has a lower defect density compared to the PEA-FASnI_3_ system, agreeing well with the higher crystallinity and stronger preferred orientation of the former. However, the thermal activation characteristics suggest that defects in the fresh devices are dominated by shallow traps, which cannot explain the large difference in the bias stress behaviors of the PEA-FASnI_3_ and FPEA-FASnI_3_ systems. Therefore, other mechanisms, in particular the redox reactions during the bias-stress process, will be investigated next.

## Defect Formation after Prolonged Operation

**Figure 5:**
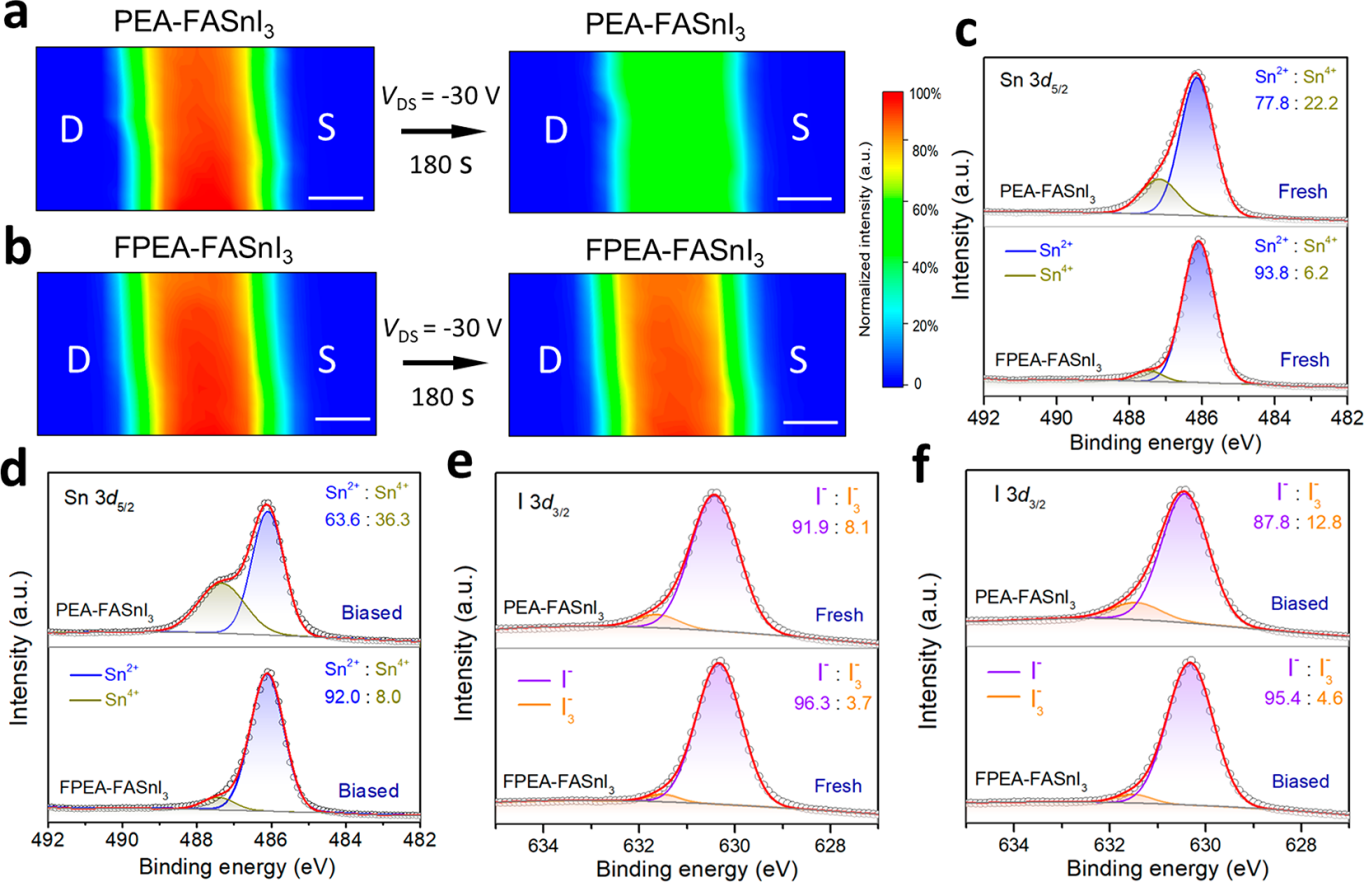
**Bias-stability mechanism characterizations**. Photoluminescence (PL) mapping was performed on lateral channels (*L* ≈ 20 μm) of FET devices based on (a) PEA-FASnI_3_ and (b) FPEA-FASnI_3_ perovskite films before and after bias-stress measurements. The bias condition is *V*_GS_ = *V*_DS_ = −30 V for 180 s. The scale bar is 10 μm. The high-resolution XPS spectra of Sn 3*d*_5/2_ core levels were performed on (c) fresh and (d) biased PEA-FASnI_3_ and FPEA-FASnI_3_ films. High-resolution XPS spectra of I 3*d*_3/2_ core levels performed on (e) fresh and (f) biased PEA-FASnI_3_ and FPEA-FASnI_3_ films.

We first conducted in situ PL mapping in the channel region to visualize the material change during the bias-stress operation. To accelerate the degradation process, a more intense bias-stress condition of *V*_GS_ = *V*_DS_ = −30 V was used. The measurement was performed in an inert environment to minimize the effects of the environmental oxygen and humidity. Figure 5a depicts the PL mapping results of the encapsulated PEA-FASnI_3_ FET device before and after electrical bias for 180 s. The PL intensity in the entire channel area exhibits a drastic drop upon biasing, and no gradient PL variation along the channel, which is related to ion migration-induced reactions,^16^ was observed. The sudden and uniform decrease of PL intensity within the channel region may be attributed to the oxidation of Sn^2+^ to Sn^4+^, which has previously been found to cause severe PL quenching in the perovskite films.^16,40^ In contrast, the PL map of the FPEA-FASnI_3_ active channel changes little after the same period of bias stress (Figure 5b), indicating the suppressed oxidation of Sn^2+^. Since the defect activities in the Sn-based perovskites are predominantly linked to the oxidation of Sn^2+^ into Sn^4+^,^41^ we employed X-ray photoelectron spectroscopy (XPS) to determine the relative ratios of Sn^2+^ species and Sn^4+^ defects in the perovskite films. First, we examine the fresh perovskite films. As shown in Figure 5c, the high-resolution Sn 3*d*_5/2_ spectra of the fresh films reveal two distinct peaks with binding energies of ∼486.2 and ∼487.4 eV, corresponding to the Sn^2+^ and Sn^4+^ states, respectively.,^16^^28^ XPS analysis shows that the Sn^4+^ oxidation state is significantly suppressed in the fresh FPEA-FASnI_3_ film with a low content of 6.2%, whereas the Sn^4+^ state increases substantially in the fresh PEA-FASnI_3_ film, reaching a content of 22.2%. These results indicate the use of the FPEAI additive can readily reduce the oxidation during the perovskite formation process.

Next, we compare the XPS results of the perovskite films after a prolonged bias stress. As shown in Figure 5d, an obvious shape deformation was observed in the Sn 3*d*_5/2_ XPS profile obtained from the biased PEA-FASnI_3_ films and the relative content of Sn^2+^ and Sn^4+^ species were measured to be 63.6% and 36.3%, respectively, showing a significant increase in Sn^4+^ defects from 22.2% in the fresh state to 36.3% after the bias stress measurement. The increase in Sn^4+^ content clearly indicates that constant bias stress with massive hole injection can promote the oxidation of Sn^2+^ to Sn^4+^, due possibly to the inherently low redox potential of the Sn^2+^/Sn^4+^ couple (∼0.15 V) in Sn-based perovskites.^21^ In fact, previous theoretical studies have predicted that endogenous Sn^2+^ oxidation is energetically favored at defective interfaces such as grain boundaries and unpassivated perovskite surfaces, especially in the presence of hole injection.^21,42^ In contrast, quite similar Sn 3*d*_5/2_ XPS spectra were observed for the FPEA-FASnI_3_ films before and after the application of the bias stress, showing a negligible increase in the Sn^4+^ content. This negligible change in Sn^4+^ content again verifies that FPEA-FASnI_3_ has fewer defects and greater oxidation resistance compared to PEA-FASnI_3_, which plausibly explains the observed superior bias stress stability and storage stability of FPEA-FASnI_3_ film-based FET devices.

In addition to Sn^2+^ oxidation, we also found the presence of relatively mild iodide oxidation, as evidenced by the changes in the core level XPS spectra of I 3*d*_3/2_. As shown in Figure 5e,f, a shoulder peak appearing at a binding energy of ∼631.5 eV is observed in both the fresh and biased films of PEA-FASnI_3_ and FPEA-FASnI_3_, clearly indicating oxidation of I^–^ to I_3_^–^.^15^ In the case of PEA-FASnI_3_, a significant content of I_3_^–^ (∼8.1%) was already detected in the fresh film, suggesting the oxidation may occur during film fabrication or storage.^43^ After the bias stress measurement, the I_3_^–^ content increased to 12.8%, suggesting that hole injection can contribute directly or indirectly to the oxidation of iodine-related defects. In striking contrast, the I_3_^–^ content in both the fresh and biased films of FPEA-FASnI_3_ remained at a low level of about 4%. A systematic comparison of the Sn^4+^ and I_3_^–^ species is shown in Figure S14, illustrating the effective suppression of Sn^4+^ and I_3_^–^ formation by the FPEAI modification.

## Density Functional Theory Calculations

**Figure 6:**
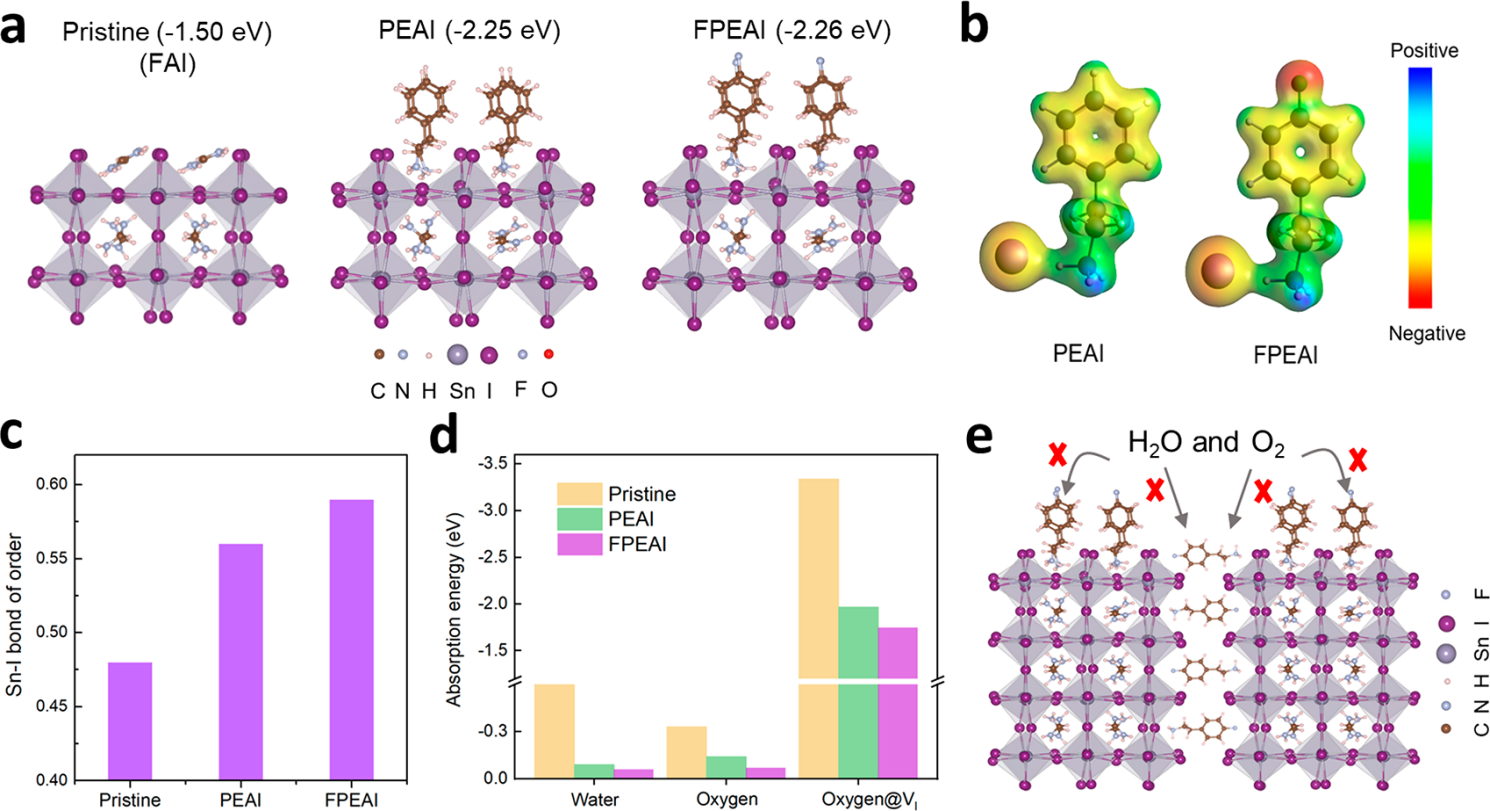
**Density functional theory (DFT) calculations**. (a) Optimized theoretical models of FASnI_3_ perovskite with FAI (pristine), PEAI and FPEAI adsorbed onto the (001) surface and DFT calculated adsorption energies are correspondingly given in the brackets. (b) Electrostatic potential (ESP) maps of PEAI and FPEAI molecules. (c) Sn–I bond orders of pristine, PEAI- and FPEAI-treated FASnI_3_ surface. (d) Adsorption energies of water, oxygen, and oxygen at V_I_ (iodine vacancy sites) on both pristine surface and the surfaces anchored with PEAI or FPEAI ligands. (e) Diagram schematically illustrating that FASnI_3_ films with FPEAI anchoring on the surface and within grain boundaries exhibit strong resistances to oxygen and water infiltration.

To gain a further understanding of the impact of PEAI and FPEAI on the crystallization and stability of the surfaces of FASnI_3_ at the atomistic level, we calculate the adsorption energy (the equation to calculate the adsorption energy can be found in the Supporting Information) and analyze the chemical bonding strength of these species with the perovskite surfaces by density functional theory (DFT) calculations. This includes investigating the adsorption of these ligands on the FASnI_3_ surface, as well as water and oxygen adsorption on the FASnI_3_ surfaces with and without PEAI and FPEAI. Initially, we investigated the absorption energy of the (001) planes of the perovskite lattice with or without the organic ligands (i.e., PEAI and FPEAI). Our results show significantly larger *E*_ads_ values (around −2.25 eV) for both PEAI and FPEAI compared to FAI (−1.50 eV), as shown in Figure 6a and Table S2, suggesting that the surface energy of the PEAI- or FPEAI-modified (001) planes were significantly reduced compared with that of pristine FASnI_3_. According to Wulff’s theorem, crystal facets with more negative absorption energy, i.e., lower surface energy, are more energetically favorable during crystal growth,^28,44^ consistent with the XRD and GIWAXS results.

To compare the differences between PEAI and FPEAI, we also calculate the electrostatic surface potential (ESP) of PEAI and FPEAI salts. Our results reveal a slightly more negative charge on the I^–^ of FPEAI compared to that of PEAI, as shown in Figure 6b. This difference arises from the substitution of the H atom with F. Consequently, the Sn–I bond becomes stronger when the FPEAI ligands are adsorbed onto the FASnI_3_ surface. This is indicated by the higher bond order (BO) and shorter bond length of Sn–I in the case of FPEAI (BO = 0.59, bond length: 2.99 Å) compared to PEAI (BO = 0.56, bond length: 3.05 Å), as depicted in Figure 6c and Figure S15. We speculate that the stronger Sn–I bond in the presence of FPEAI contributes to a lower trap density in the FPEAI-treated films than in the PEAI-treated films, as experimentally measured.

Finally, we investigate the stability of the FASnI_3_ surface under moisture and oxygen atmospheres by calculating the adsorption energies of water and oxygen on both the pristine surface and surfaces anchored with PEAI or FPEAI ligands. The adsorption energies, along with the corresponding structures, are given with details in the Supporting Information (Figures S16 and 17, and Table S3 and S4). Figure 6d illustrates that the adsorption of water on top of the ligands (PEAI: −0.09 eV of *E*_ads_; FPEAI: −0.06 eV of *E*_ads_) is evidently unfavorable compared to the configurations where water interacts with pristine surfaces FASnI_3_ (most favorable site: −0.60 eV of *E*_ads_). This suggests that the addition of both ligands plays a role in passivating the favorable water adsorption sites. Furthermore, FPEAI ligand exhibits greater resistance to water, as evidenced by the weaker bond between F and H from water (bond length: 2.72 Å, BO: 0.06) compared to the bond between H from PEAI and O from water (bond length: 2.42 Å, BO: 0.08), as shown in Figure S16 and Table S3. The enhanced water resistance induced by the FPEAI ligands has also been experimentally demonstrated through contact angle measurements. As depicted in Figure S18, the perovskite films of FPEA-FASnI_3_ exhibited superior hydrophobicity, as evidenced by a notably larger contact angle in comparison to PEA-FASnI_3_ films. Similarly, FPEAI is more likely to repel oxygen with an *E*_ads_ of −0.07 eV, whereas PEAI shows an *E*_ads_ of −0.14 eV when oxygen is adsorbed on top. This can be attributed to the weak interaction between oxygen and FPEAI (bond length: 2.97 Å, BO: 0.06, in Figure S16), in contrast to the stronger O–H (PEAI) bond with a length of 2.51 Å and BO of 0.11. This preference results from oxygen’s affinity for interacting with the more positive H from PEAI rather than the negative F from FPEAI. Most notably, the adsorption of oxygen on the iodine vacancy (V_I_) site of the FPEAI-treated surface is evidently less favorable than that on the PEAI-treated surface. This is crucial because oxygen can obtain charges from the V_I_ to form superoxide and peroxide,^45^ potentially leading to the oxidation of Sn^2+^. Therefore, compared to PEAI, FASnI_3_ surfaces anchored with FPEAI exhibit improved resistance not only to water but also to oxygen species, and the prevention effect is more pronounced in the presence of iodine defects. As such, the FPEAI modification can better prevent the oxidation of Sn^2+^, ultimately enhancing its stability against moisture and oxygen atmospheres. The enhanced stability of the FPEA-FASnI_3_ films is further confirmed with XRD measurements, as shown in Figure S19.

Combining all of the observations, we can now explain the dramatic difference in the bias-stress stability of the PEA-FASnI_3_ and FPEA-FASnI_3_ transistors. First, the FPEAI molecules contribute to better crystallinity, preferential orientation, and decreased structural defects compared to the pristine FASnI_3_ and PEA-FASnI_3_ films. The defect reduction can readily help to the lower initial concentration of the Sn^4+^ species,^21^ and further suppress the reaction between the Sn^2+^ species and injected holes during bias stress. Moreover, with its stronger capability of repelling water and oxygen, the FPEAI molecule (Figure 6e) can help to reduce unintentional trapping of water and oxygen molecules in the perovskite films during device fabrication. It has been studied that the presence of oxygen and moisture could induce a series of reactions and accelerate the degradation of FASnI_3_ perovskites.^46^ The observed I_3_^–^ species in our XPS spectra also point to such chain reactions, promoted by a synergistic effect of defects, water, oxygen, and electrical bias stress. Therefore, the stability enhancement by FPEAI is achieved by minimizing the water, oxygen, and defect content in both the fabrication and device operation stages.

In summary, we have demonstrated and systematically investigated the role of phenethylammonium iodide additives in enhancing the performance and stability of 3D pure tin-based FETs. The combined theoretical and experimental study reveals that, on the one hand, the phenethylammonium molecules, with and without fluorination, can both retard the perovskite formation process and consequently enhance crystal growth with strong preferential orientation; while on the other hand, the fluorinated phenethylammonium molecules are much more effective in reducing structural defects, suppressing oxidation of Sn^2+^ and blocking oxygen and water involved defect reactions. Promisingly, the optimized FPEA-FASnI_3_ FETs exhibit high field-effect mobility, high on/off current ratio, negligible hysteresis, and excellent operational stability and long-term stability at the same time. The study sheds light on the fundamental principles and material designs for realizing stable and high-performance lead-free perovskite FETs.

## Supporting Information Available

Materials used, methods of perovskite precursor preparation, solution-based perovskite film coating, device fabrication and characterization, instrumentation techniques, perovskite film characterization, and details of DFT calculations (PDF)
