# Supplementary material for: High-Mobility and Bias-Stable Field-Effect Transistors Based on Lead-Free Formamidinium Tin Iodide Perovskites
Source: ACS Energy Lett. 2023 Oct 2;8(10):4496–505. doi: 10.1021/acsenergylett.3c01400 (PMC10580314; doi:10.1021/acsenergylett.3c01400)
Supplement: Supplementary file 1 — nz3c01400_si_001.pdf [file nz3c01400_si_001.pdf]

Supporting Information

High-Mobility and Bias-Stable Field-Effect Transistors Based on Lead-Free Formamidinium Tin Iodide Perovskites

Zhiwen Zhou[1], Qihua Li[2], Mojun Chen[3], Xuerong Zheng[1], Xiao Wu[4], Xinhui Lu[4], Shuxia Tao[2], and Ni Zhao[1]*

[1]Department of Electronic Engineering, The Chinese University of Hong Kong, Shatin 999077, New Territories, Hong Kong SAR, China;

[2]Materials Simulation & Modelling, Department of Applied Physics, Eindhoven University of Technology, 5600 MB Eindhoven, The Netherlands;

[3]Smart Manufacturing Thrust, Systems Hub, The Hong Kong University of Science and Technology, Guangzhou 511458, China;

[4]Department of Physics, The Chinese University of Hong Kong, Shatin 999077, Hong Kong, Hong Kong SAR, China;

Correspondence and requests for materials should be addressed to Prof. Ni Zhao (nzhao@ee.cuhk.edu.hk).

EXPERIMENTAL METHODS

Materials: Formamidinium iodide (FAI, 99.9%), phenethylammoniumiodide (PEAI), and 4-fluoro-phenethylammonium iodide (4-FPEAI) was purchased from GreatCell Solar Materials. Tin(II) iodide (SnI_2, 99.99%) and tin(II) fluoride (SnF_2, 99%) were purchased from Sigma-Aldrich. *N,N*-dimethylformamide (DMF, anhydrous, 99.8%), dimethyl sulfoxide (DMSO,

anhydrous, 99.7+%) and chlorobenzene (CB, anhydrous 99.8%) were bought from Hong Kong Labware Co. Ltd. All the chemicals and solvents were used as received without further purification.

Perovskite precursor preparation: The perovskite precursors were prepared by mixing the mother solutions of FAI (0.8 M, dissolved in DMF), SnI_2 (0.8 M, dissolved in DMF, with 10 mol% of SnF_2 as the additive), PEAI (0.8 M, dissolved in DMF) and FPEAI (0.8 M, dissolved in DMF) at varied volume ratios and the obtained mixed precursors were further diluted with DMF and DMSO solvents to get the target concentrations with a final 4:1 volume ratio of DMF: DMSO. For example, for 0.2 M of the pristine $FASnI_3$ precursor, equal volume rations of FAI (0.8 M) and SnI_2 (0.8 M) were mixed and diluted with the DMF and DMSO solvents. For the organic ligands incorporated $FASnI_3$, the corresponding amount of FAI were replaced by PEAI or FPEAI. For instance, to get an optimized solution of 0.2 M $FASnI_3$ with 9.1 % volume ratio of FPEAI, different volume ratios of FPEAI, FAI and SnI_2 (FPEAI: FAI: SnI_2 = 1: 11: 12) were mixed and further diluted with DMF and DMSO solvents. Notably, all the precursor solutions were stirred at room temperature for 20 min before use. The precursor solutions were prepared in a nitrogen glove box with oxygen and water concentrations less than 10 ppm.

Device fabrication and characterization: Perovskite field-effect transistors (FETs) were fabricated on cleaned Si/SiO_2 substrates in bottom-gate and bottom-contact device configurations. The thickness of SiO_2 dielectric layer is 100 nm with an areal capacitance of ~34 nF/cm^2. The substrates were cleaned by standard cleaning procedures including successive sonication in deionized water, ethanol, acetone, and 2-propanol for 15 min, respectively. The

cleaned substrates were further put into a vacuum chamber for depositing 5 nm Cr/40 nm Au as the source and drain electrodes. The channel length (L) and width (W) are around 100 μm and 1500 μm, respectively. The substrates with electrodes were then treated with UV-ozone for 30 min to remove organic residues and improve surface wettability. After the UV-ozone treatment, the substrates were quickly transferred inside the glove box for the deposition of perovskite films. The perovskite channels were spin-coated at 5000 rpm for 60 s and 100 μl of chlorobenzene was dropped gently on the substrates at 10th s. Subsequently, the perovskite films were annealed at 95 °C for 10 min. As for the FET device characterizations, the transfer curves, output curves and bias stress stability tests were conducted by Keithley 2612 source meter in dark conditions and at room temperature within a nitrogen-filled glove box. Temperature-dependent charge transport measurements were performed using a Desert Cryogenics low-temperature probe station and Keithley 4200 semiconductor characterization systems under vacuum conditions. The measured mobility in the linear and saturated regimes were extracted from the slopes of I_{DS} *vs* V_{GS} and $I_{DS}^{1/2}$ *vs* V_{GS} plots, respectively, according to equations of $I_{DS} = W/L \cdot C_i \cdot \mu \cdot (V_{GS}-V_{TH}) \cdot V_{DS}$ in the linear regime and $I_{DS} = W/(2L) \cdot C_i \cdot \mu \cdot (V_{GS}-V_{TH})^2$ in the saturation regime.

Perovskite film characterization: The surface morphology of perovskite films was checked using a field-emission scanning electron microscope (SEM, S4800 Hitachi). An atomic force microscope (AFM, Dimension Icon) purchased from Bruker company was used to characterize the grain size and film thickness of perovskite layers under inert atmosphere. The phase identification and crystallinity of perovskite films were characterized by using an XRD (Rigaku SmartLab) with Cu Kα radiation at 40 kV and 80 mA. GIWAXS measurements were

performed with a Xeuss 2.0 SAXS/WAXS laboratory beamline with a Cu X-ray source (8.05 eV, 1.54 Å) and a small incident angle of 0.3° was used during the GIWAXS measurements. Photoluminescence (PL) spectra and in-situ PL mapping under voltage bias were carried out by Horiba PL spectrometer with a dynamic motion stage. The average power of the laser at the focus point was around 1 μW during the PL mapping tests. Time-resolved PL decays were conducted with a FLS 980 spectrofluorometer (Edinburgh Instruments), excited by 465 nm laser with excitation power of 10 μW. The XPS measurements were performed using an X-ray photoelectron spectrometer (PHI 5600 multi-technique systems, Physical Electronics).

Details of DFT calculations

The DFT calculations were performed using the Vienna *ab Initio* Simulation Package (VASP).[1] The calculations were performed with the generalized gradient approximation as proposed by Perdew, Burke, and Ernzerhof (PBE), with energy and force convergence parameters of 1×10^{-5} eV and 1×10^{-2} eV/Å, respectively.[2,3] The long-range dispersive interactions between adsorbates (ligands, water, and oxygen) and the surface of $FASnI_3$ (with and without ligands) were corrected by DFT-D3 scheme.[4] For the geometry optimization, 9-layer FAI-terminated and SnI_2-terminated $FASnI_3$ slabs were used (2 × 2 supercell in ab plane), with 15 Å thick vacuum on the top of upper surface as well as below the bottom surface, respectively. To simulate the adsorption of ligands, FAI, PEAI and FPEAI were put on top of the SnI_2-terminated slab. The calculations were performed with a 3 × 3 × 1 *k*-point mesh and a kinetic energy cut-off of 500 eV. The adsorption energies were calculated as the following: $E_{ads} = (E_{ligands/FASnI3} - E_{FASnI3} - n\cdot E_{ligands})/n$, where ligands represent PEAI/FPEAI, and n represents the number of ligands on the surface of supercell. The electrostatic potential distribution of PEAI and FPEAI was

evaluated using a TZP basis set incorporated in the Amsterdam Density Functional (ADF) program.[5] To assess the bond strength between adsorbates (ligands, water, and oxygen) and the surface of $FASnI_3$ (both with and without ligands), the bond order was determined using the Density Derived Electrostatic and Chemical (DDEC6) method.[6] The bond order calculation allows for the quantification of covalent or hydrogen bonding strength, with larger bond order values indicating stronger bonding interactions.[7,8]

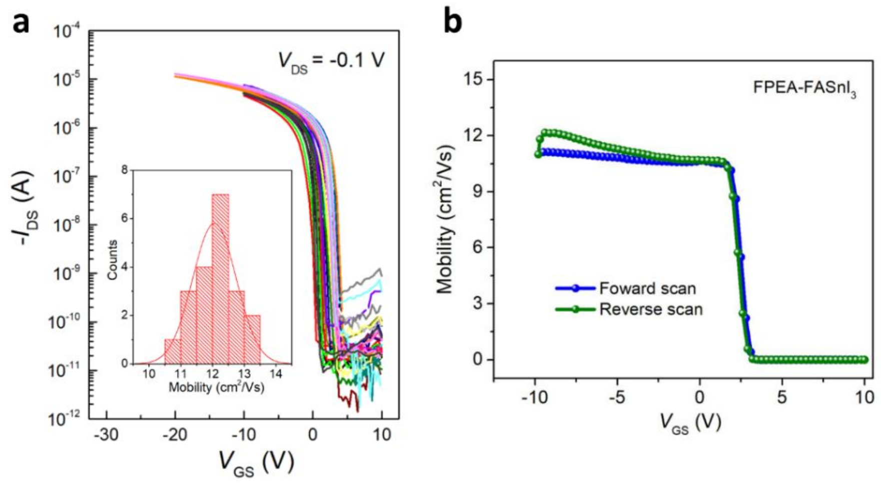

Figure S1. (a) Statistic transfer curves of 20 FPEA-$FASnI_3$ devices as well as the statistic histogram of carrier mobilities that measured in the linear region. (b) The corresponding mobility-gate voltage dependency curve of one typical device of them. The measured average mobility is as high as 12.1 cm^2/Vs, with a small standard deviation of 0.5 cm^2/Vs.

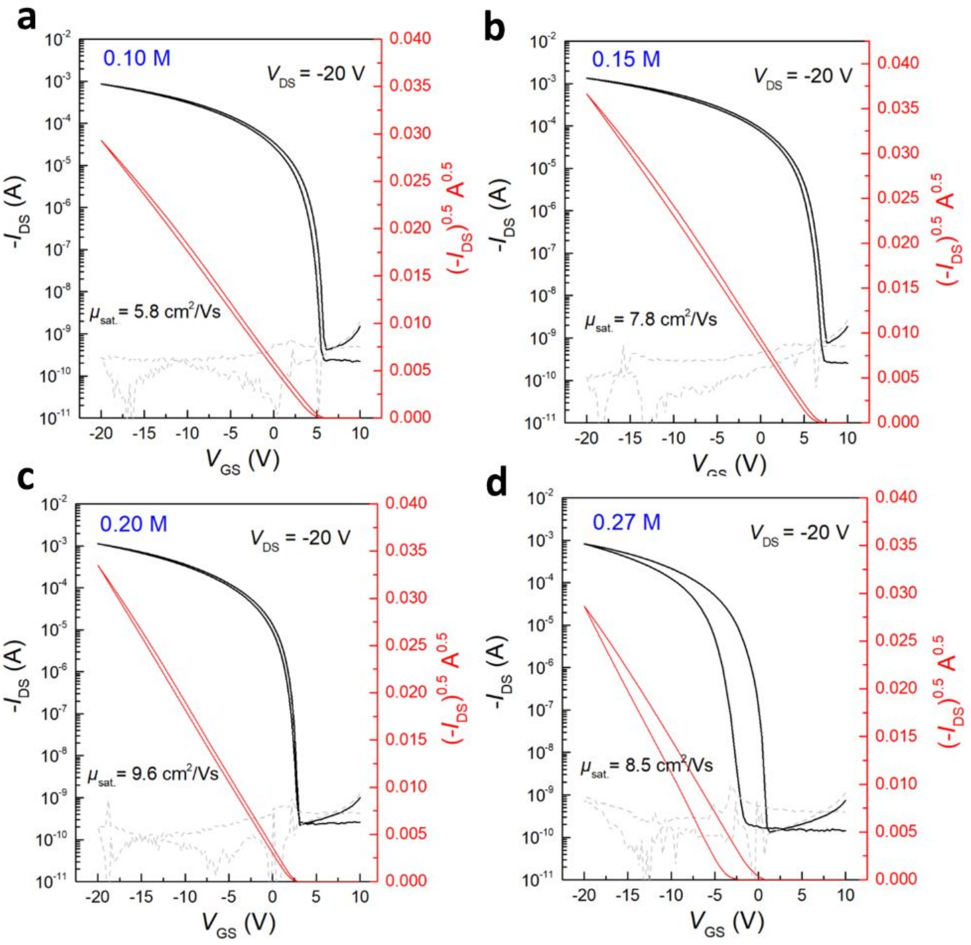

Figure S2. Saturated transfer curves of typical FPEA-$FASnI_3$ devices that were fabricated by different perovskite precursor concentrations. (a) 0.1 M; (b) 0.15 M; (c) 0.20 M; (d) 0.27 M. All the perovskite films were spin coated by using a same spin speed of 5000 rpm.

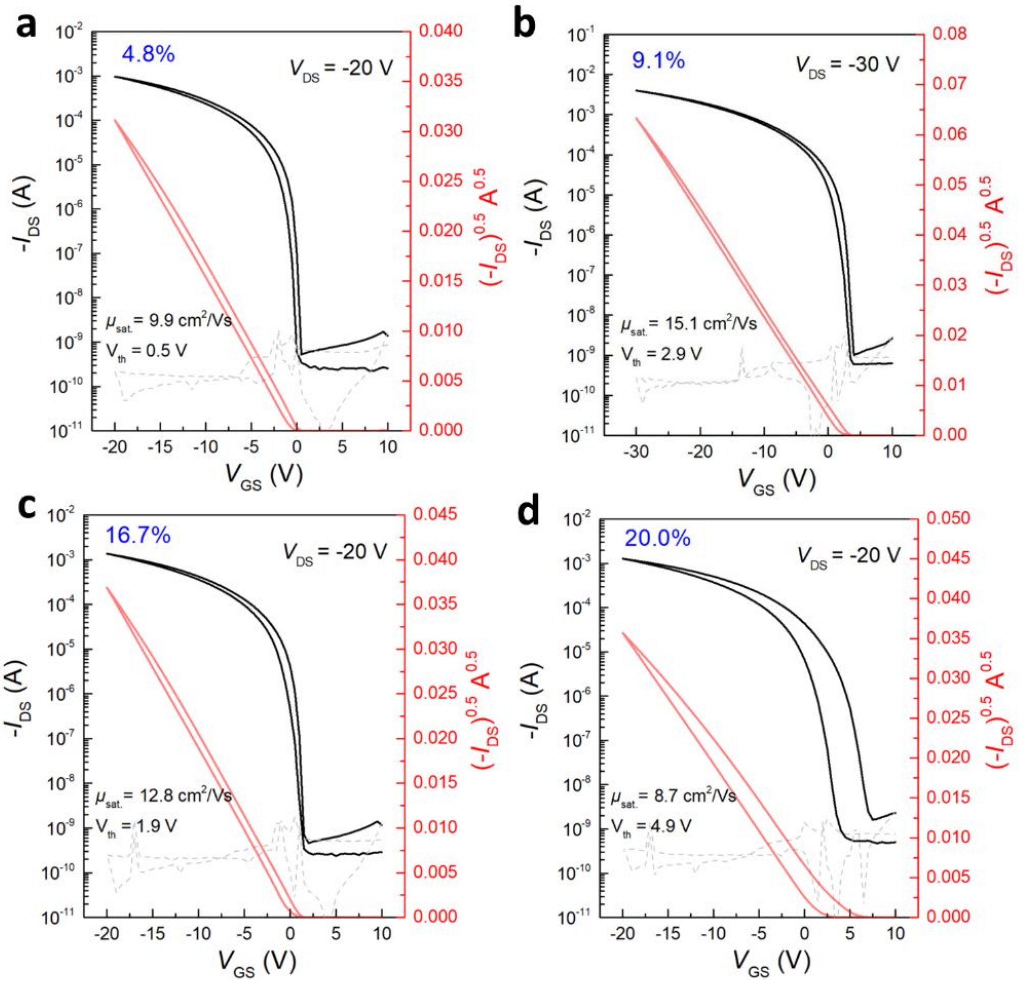

Figure S3. Saturated transfer curves of typical FPEA-$FASnI_3$ devices that were fabricated by adding different volume ratios of FPEA solution with respect to the volume of FAI mother solution into perovskite precursors. (a) 4.8%; (b) 9.1%; (c) 16.7%; (d) 20.0%. All the perovskite films were spin coated by using a same spin speed of 5000 rpm.

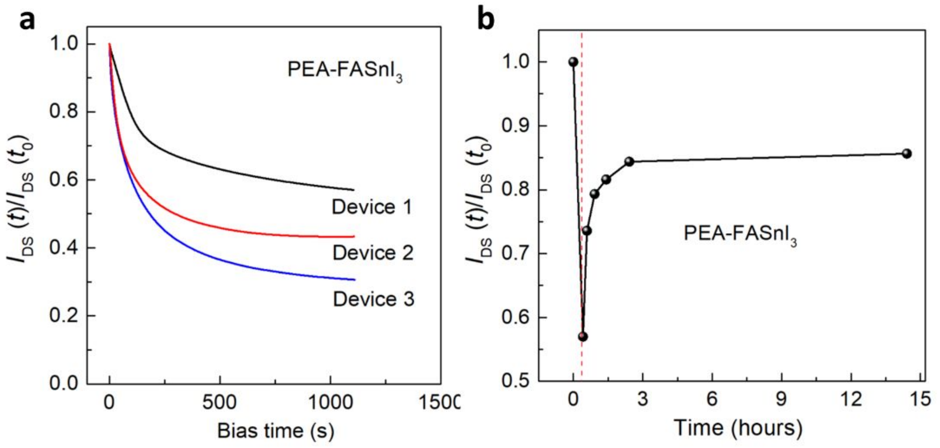

Figure S4. (a) Bias-stress stability measurements performed on three different PEA-$FASnI_3$ FETs. A constant bias condition of V_{GS} = -10 V and V_{DS} = -1 V was used during the bias-stress measurements. (b) The I_{DS} current evaluation is used to track the electrical degradation and recovery of the transistor (Device 1) during the stress and recovery periods. The dotted line in (b) represent the boundary between the stress and recovery periods.

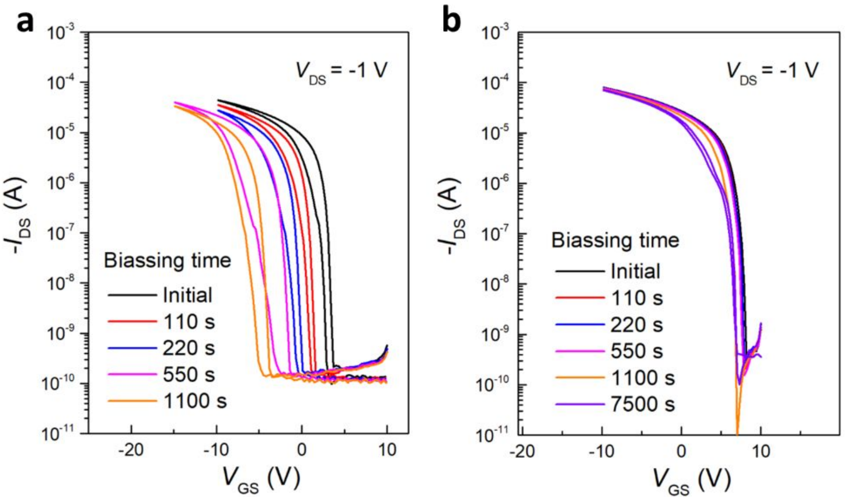

Figure S5. Operational stability of $FASnI_3$-based perovskite transistors. Transfer curves of one typical (a) PEA-$FASnI_3$ device and (b) FPEA-$FASnI_3$ device. All the devices were properly encapsulated and tested under a negative-bias condition (V_{GS} = -10 V; V_{DS} = -1 V) for different durations in the glove box.

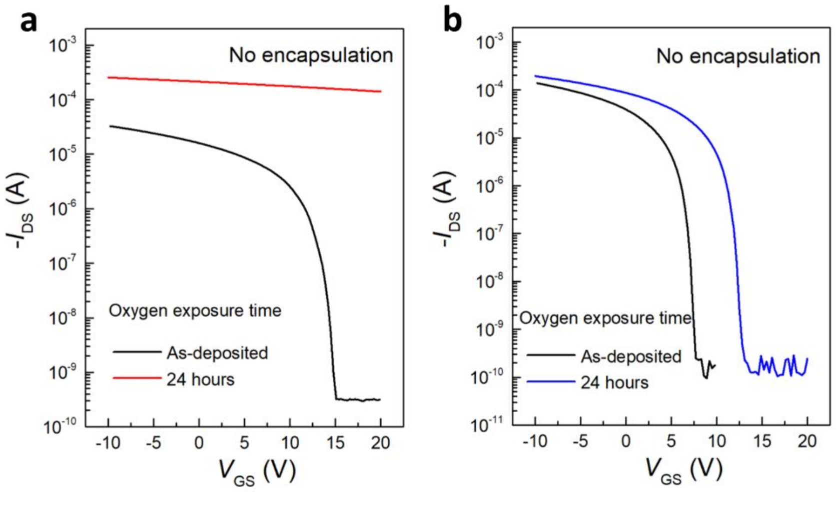

Figure S6. Oxygen exposure stability of $FASnI_3$-based perovskite transistors. Transfer curves of (a) PEA-$FASnI_3$ devices and (b) FPEA-$FASnI_3$ devices. All the devices were barely tested

in the glove box (Oxygen level ~25 ppm; H_2O level ~1ppm).

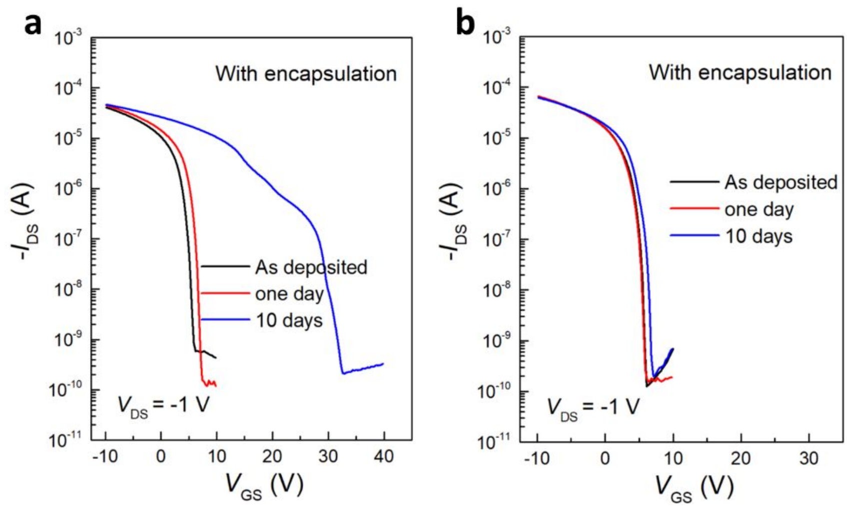

Figure S7. Device stability of $FASnI_3$-based perovskite transistors with PIB glass encapsulation. Transfer curves of (a) PEA-$FASnI_3$ devices and (b) FPEA-$FASnI_3$ devices. All the devices were tested in the glove box (Oxygen level ~25 ppm; H_2O level ~1ppm).

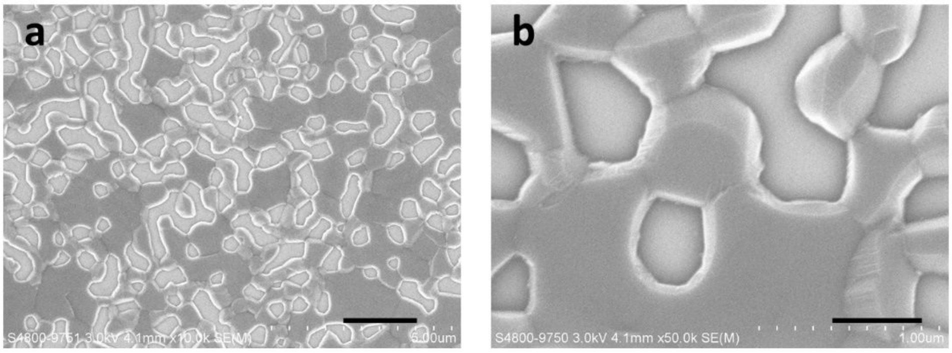

Figure S8. SEM images of pristine $FASnI_3$ films prepared by spin coating (5000rpm/60s) with a precursor concentration of 0.2 M. The scale bars in (a) and (b) are 2 μm and 0.5 μm, respectively.

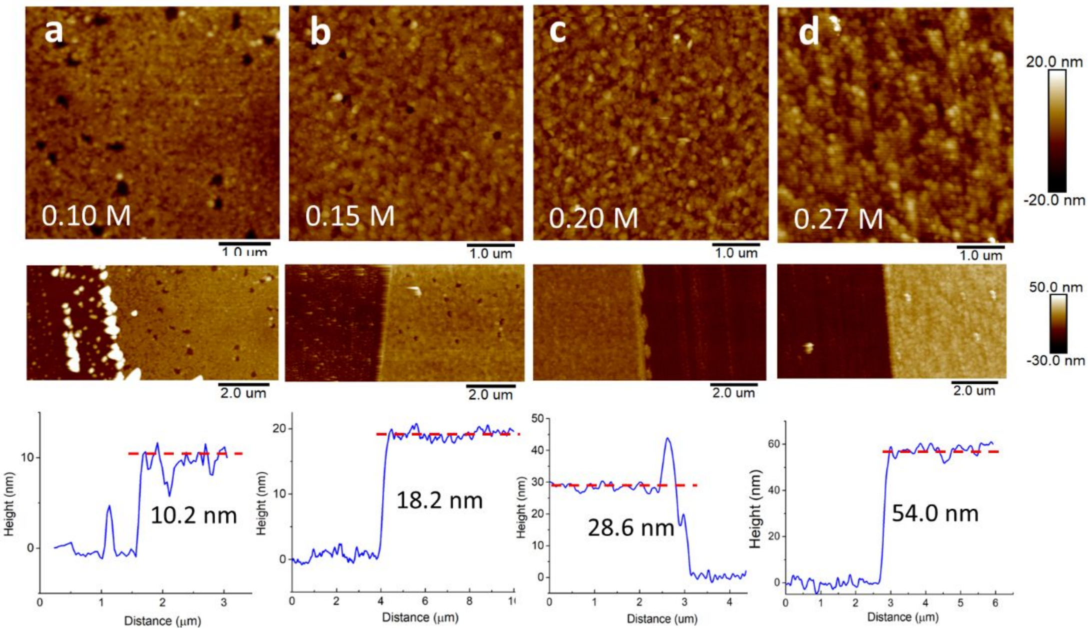

Figure S9. AFM surface morphology and film thickness of FPEA-$FASnI_3$ perovskites that were fabricated by different precursor concentrations. (a) 0.1 M; (b) 0.15 M; (c) 0.20 M; (d) 0.27 M. All the perovskite films were spin coated by using a same spin speed of 5000 rpm.

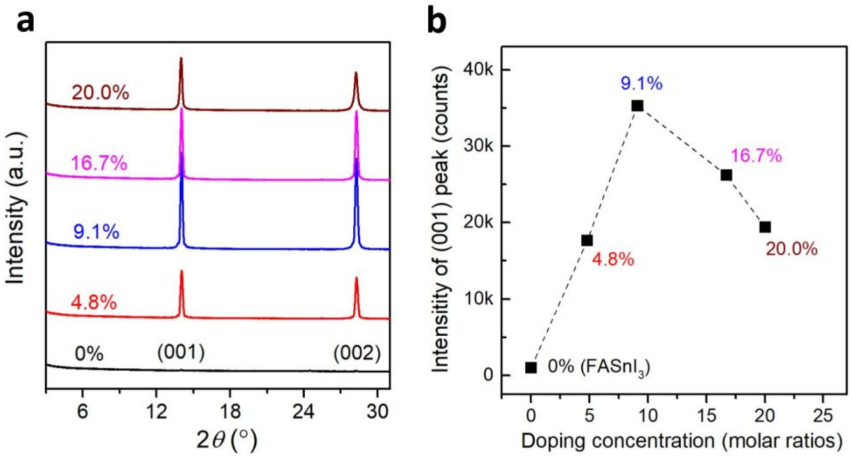

Figure S10. Crystal structure variation as a function of the FPEAI concentration. (a) XRD patterns and (b) the corresponding (001) peak intensity of the FPEA-$FASnI_3$ perovskite films with different molar ratios of FPEAI in the perovskite precursor. All the films were fabricated and tested in the same condition.

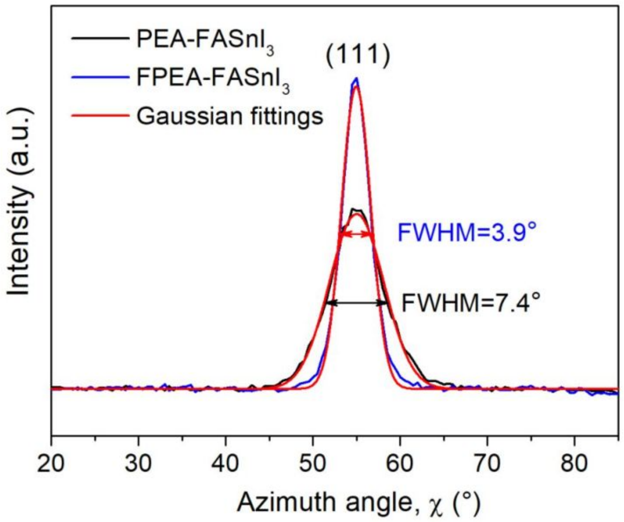

Figure S11. Angular intensity profiles of PEA- and FPEA-$FASnI_3$ films along the azimuth angle at q_z = 1.62 Å^{-1}. The full-with at the half-maximum (FWHM) values at the (111) peak position were obtained by fitting the peak profiles with a Gaussian function.

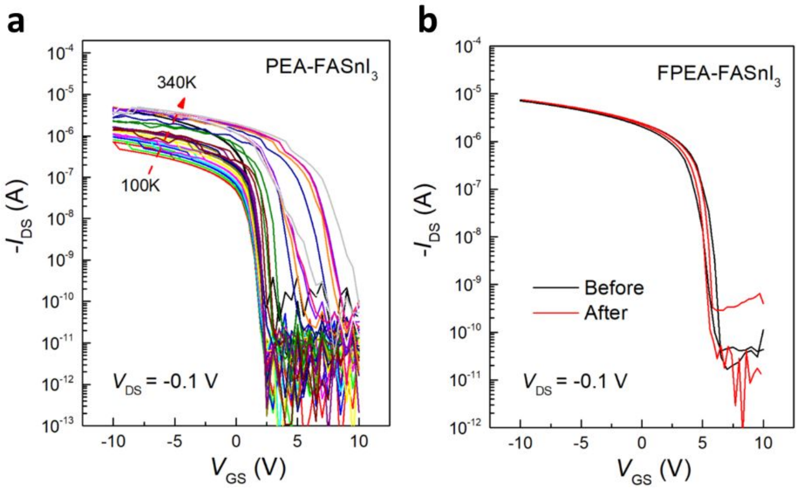

Figure S12. (a) Temperature-dependent electrical transfer curves measured on one typical PEA-$FASnI_3$ FET. (b) Transfer cures of the FPEA-$FASnI_3$ transistor before and after a temperature change circle from the room temperature (~291K) to the low temperature (110 K).

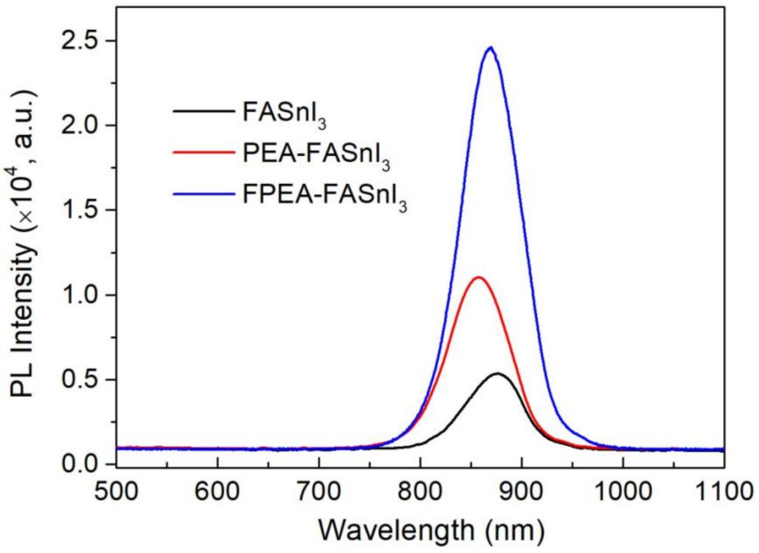

Figure S13. Steady-state PL spectra of the pristine $FASnI_3$, PEA-$FASnI_3$ and FPEA-$FASnI_3$ perovskite films.

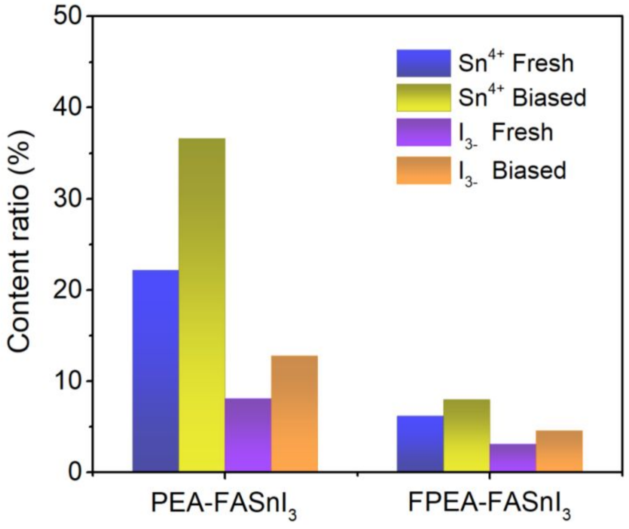

Figure S14. The content of Sn^{4+} and I_3^- species in PEA-$FASnI_3$ and FPEA-$FASnI_3$ films under fresh and biased conditions.

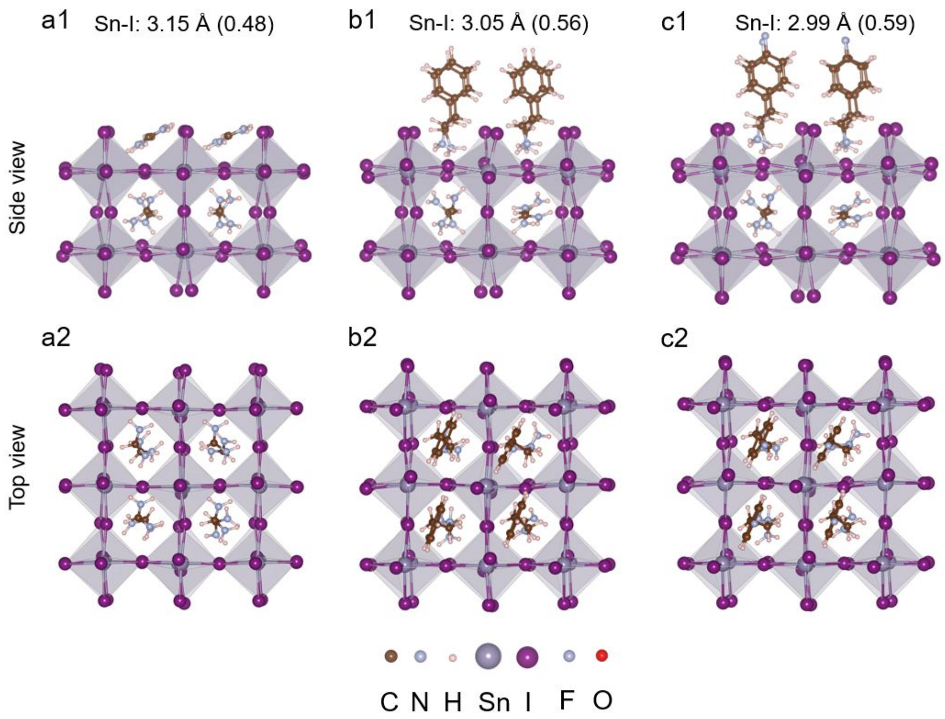

Figure S15. The adsorption of FAI, PEAI and FPEAI on the surface of $FASnI_3$. Optimized structures of (a) FAI, (b) PEAI and (c) FPEAI on the surface of $FASnI_3$, respectively. The values and the values in the bracket are the bond length and bond order (BO) of Sn-I bond, respectively.

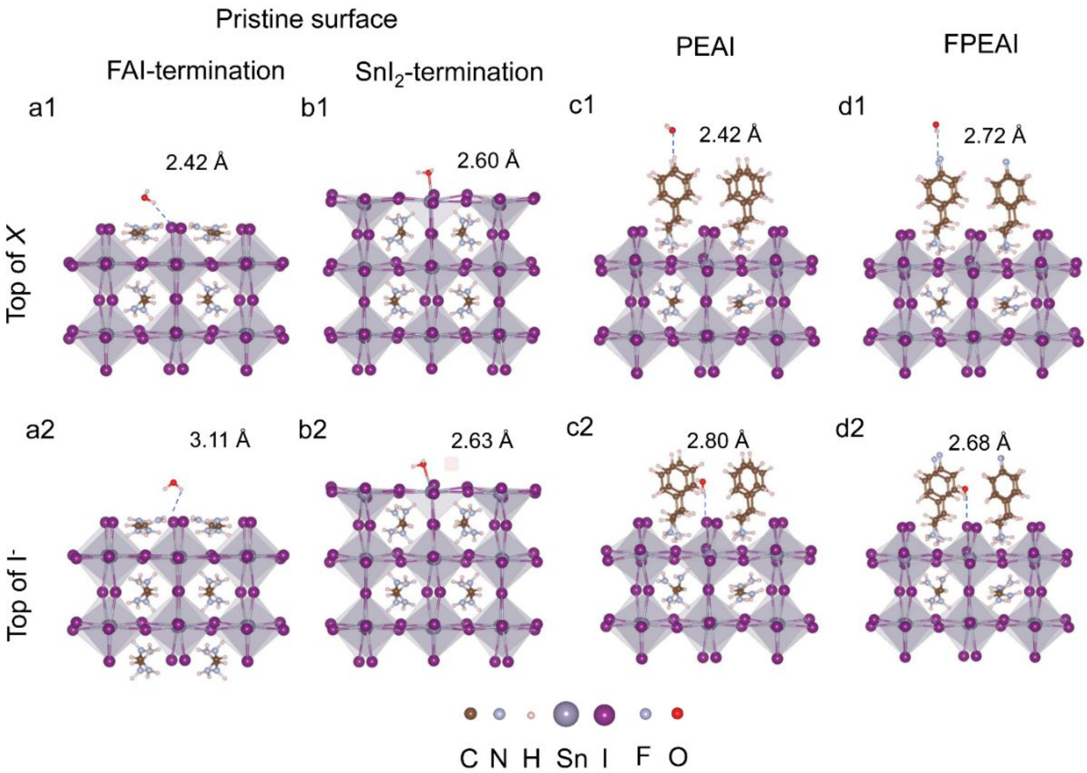

Figure S16. The adsorption of water on the surface of pristine $FASnI_3$, the $FASnI_3$ surface with PEAI and FPEAI. Optimized structures of water adsorption on (a) FAI-termination, (b) SnI_2-termination, the surface with (c) PEAI and with (d) FPEAI of $FASnI_3$ at the sites (1) top of X ($X=FA^+$, Sn^{2+}, H and F) and top of I^-, respectively. The values inside are the distances between water and $FASnI_3$/ligands.

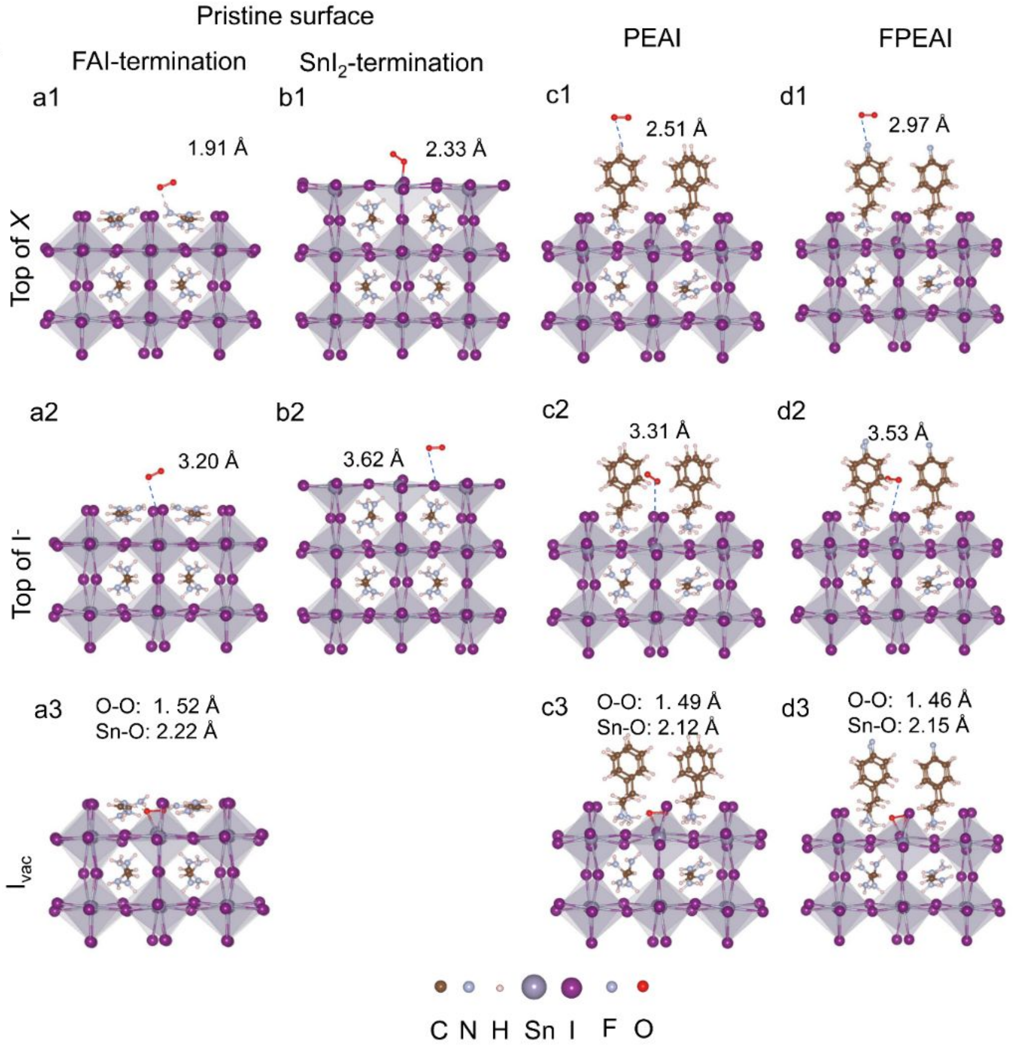

Figure S17. The adsorption of oxygen on the surface of pristine $FASnI_3$, the $FASnI_3$ surface with PEAI and FPEAI. Optimized structures of oxygen adsorption on (a) FAI-termination, (b) SnI_2-termination, the surface with (c) PEAI and with (d) FPEAI of $FASnI_3$ at the sites (1) top of X (X=FA^+ of FAI-termination, Sn^{2+} of SnI_2- termination, H of PEAI and F of FPEAI), (2) top of I^- and (3) V_I (iodine vacancy) respectively. The values inside are the distances between water and $FASnI_3$/ligands.

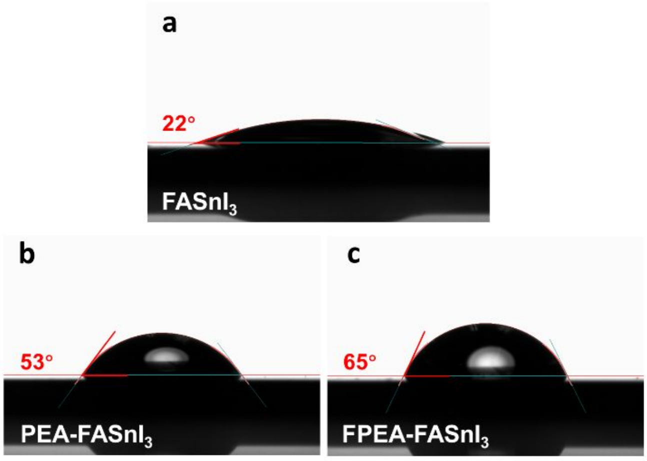

Figure S18. Photographs of the contact angles of water droplets on the surface of (a) pristine $FASnI_3$, (b) PEA-$FASnI_3$ and (c) FPEA-$FASnI_3$ perovskite films. Note that all perovskite films were fabricated with the same precursor concentration of 0.2 M.

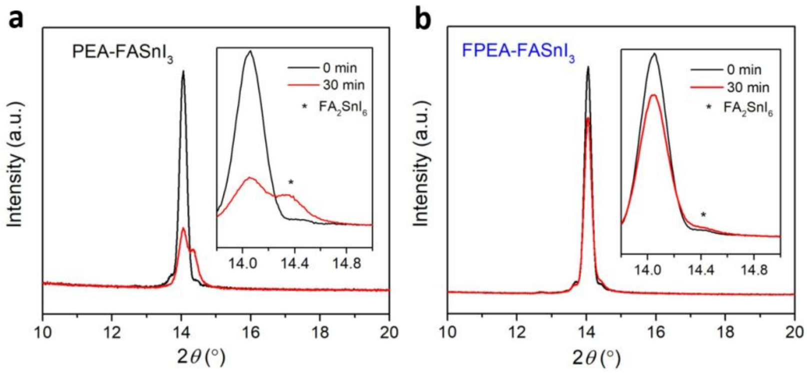

Figure S19. Intensity evolution of the (001) XRD peak of (a) PEA-$FASnI_3$ and (b) FPEA-$FASnI_3$ perovskite films upon exposure to ambient environment (R.H. 68% ±7.5%) at room temperature. Inset figures are the magnification of crystal peaks around 14° diffraction position and peaks labelled with the star symbol indicate the degradation phase of FA_2SnI_6.

Table S1. Summarized performance evolution of lead-free Sn-based organic-inorganic hybrid

perovskite FETs.

	Semiconductor /Dielectric materials /Contact	Device structure	Deposition/ treatment	μ_h [cm^2/Vs]		SS [V/dec]	I_{on} $/I_{off}$	Year	Ref.
				$\mu_{lin.}$	$\mu_{sat.}$				
2D	$PEA_2SnI_4/SiO_2/Au$	BGBC	NH_3I–SAM-treatment	/	2.5	~1.8	~10^6	2016	[9]
	$PEA_2SnI_4/SiO_2/Au$	BGTC	NH_3I–SAM-treatment	/	7.1	~1.1	~10^6	2016	
	PEA_2SnI_4/Cytop/Au	TGTC	NH_3I–SAM-treatment $/MoO_x$	/	12.0	~0.8	~10^6	2016	
	$PEA_2SnI_4/SiO_2/Au$	BGTC	One-step spin-coating	/	1.0	~5.0	~10^3	2017	[10]
	PEA_2SnI_4/PVA/Cl-PVP/Au	BGTC	Polymer dielectric	/	0.3	~7.9	~10^3	2019	[11]
	$PEA_2SnI_4/SiO_2/Au$	BGTC	Semi-CNTs mixed with precursor	/	1.5	~2.1	~10^5	2019	[12]
	$(4Tm)_2SnI_4/SiO_2/Au$	BGTC	/	/	1.8	~10	~10^5	2019	[13]
	$PEA_2SnI_4/SiO_2/Au$	BGTC	Precursor optimization /iodine vacancy passivation	/	3.5	~0.8	~10^6	2020	[14]
	$PEA_2SnI_4/SiO_2/Au$	BGTC	Binary solvent engineering	/	3.8	~2.1	~10^6	2020	[15]
	$PEA_2SnI_4/SiO_2/Au$	BGTC	Lewis-base urea mixed with precursor	/	4.2	~1.9	~10^5	2021	[16]
	$(TT)_2SnI_4$	BGTC	/	/	6.5	~5.4	~10^5	2021	[17]
	$(STm)_2SnI_4$	BGTC	/	/	1.4	~7.0	~10^6	2021	[18]
2D/3D Hybrid	$FASnI_3/(PEA)_2SnI_4$/PMMA $/Al_2O_3/Au$	TGBC	Dielectric optimization /2D material mixture	/	0.2	~2.0	~10^4	2021	[19]
	$FASnI_3/(PEA)_2SnI_4/HfO_2/Au$	BGBC	2D material mixture	/	11.8	~0.1	~10^8	2023	[20]
3D	$MASnI_3/HfO_2/Au$	BGTC	/	/	1.3	~0.6	~10^4	2022	[21]
	$FASnI_3/SiO_2/Au$	BGBC	F-PEAI passivation	12.1	13.5	~0.1	~10^7	This work	

Channel semiconductors are solution-processed unless mentioned otherwise. μ_h indicates the hole carrier mobility. $\mu_{lin.}$ and $\mu_{sat.}$ mean the averaged carrier mobility measured in the linear and saturated region, respectively. SS is the abbreviation of subthreshold swing, while I_{on}/I_{off} represents the ratio of the on- and off-state current.

Table S2. Adsorption energies (E_{ads}) and bond order (BO) of FAI/ligands on SnI_2-terminated surface.

Adsorbates:	E_{ads}	BO
FAI	-1.50	0.48
PEAI	-2.25	0.56
FPEAI	-2.26	0.59

Table S3. Adsorption energies (E_{ads}) and bond order (BO) of water on different configurations of $FASnI_3$ surface.

	Top of X		Top of I	
Site:	E_{ads}	BO	E_{ads}	BO
FAI-termination	-0.21	0.28	-0.12	0.13
SnI_2-termination	-0.60	0.50	-0.41	0.50
PEAI	-0.09	0.08	-0.41	0.39
FPEAI	-0.06	0.06	-0.37	0.31

(X=FA^+ of FAI-termination, Sn^{2+} of SnI_2-termination, H of PEAI and F of FPEAI)

Table S4. Adsorption energies (E_{ads}) and bond order (BO) of oxygen on different configurations of $FASnI_3$ surface.

	Top of X		Top of I		Defective	
Site:	E_{ads}	BO	E_{ads}	BO	E_{ads}	BO
FAI-termination	-0.33	0.30	-0.12	0.16	-	-
SnI_2-termination	-0.25	0.26	-0.15	0.15	-3.34	1.66
PEAI	-0.14	0.11	-0.13	0.1	-1.97	1.34
FPEAI	-0.07	0.06	-0.11	0.07	-1.75	1.28

(X=FA^+ of FAI-termination, Sn^{2+} of SnI_2-termination, H of PEAI and F of FPEAI; Defective site indicates the iodine vacancy site, V_I.)

References

(1) Kresse, G.; Furthmüller, J. Efficient iterative schemes for ab initio total-energy calculations using a plane-wave basis set. *Phys. Rev. B* **1996**, *54* (16), 11169.

(2) Kresse, G.; Hafner, J. Ab initio molecular dynamics for liquid metals. *Phys. Rev. B* **1993**,

47 (1), 558.
(3) Perdew, J. P.; Burke, K.; Ernzerhof, M. Generalized gradient approximation made simple. *Phys. Rev. Lett.* **1996**, *77* (18), 3865.
(4) Grimme, S.; Ehrlich, S.; Goerigk, L. Effect of the damping function in dispersion corrected density functional theory. *J. Comput. Chem.* **2011**, *32* (7), 1456-1465.
(5) Kim, J. H.; Kim, Y. R.; Kim, J.; Oh, C. M.; Hwang, I. W.; Kim, J.; Zeiske, S.; Ki, T.; Kwon, S.; Kim, H. Efficient and Stable Perovskite Solar Cells with a High Open-Circuit Voltage Over 1.2 V Achieved by a Dual-Side Passivation Layer. *Adv. Mater.* **2022**, *34* (41), 2205268.
(6) Limas, N. G.; Manz, T. A. Introducing DDEC6 atomic population analysis: part 2. Computed results for a wide range of periodic and nonperiodic materials. *RSC Adv.* **2016**, *6* (51), 45727-45747.
(7) Li, Q.; Chen, Z.; Tranca, I.; Gaastra-Nedea, S.; Smeulders, D.; Tao, S. Compositional effect on water adsorption on metal halide perovskites. *Appl. Surf. Sci.* **2021**, *538*, 148058.
(8) Li, Q.; Rellán-Piñeiro, M.; Almora-Barrios, N.; Garcia-Ratés, M.; Remediakis, I. N.; López, N. Shape control in concave metal nanoparticles by etching. *Nanoscale* **2017**, *9* (35), 13089-13094.
(9) Matsushima, T.; Hwang, S.; Sandanayaka, A. S.; Qin, C.; Terakawa, S.; Fujihara, T.; Yahiro, M.; Adachi, C. Solution-processed organic–inorganic perovskite field-effect transistors with high hole mobilities. *Adv. Mater.* **2016**, *28* (46), 10275-10281.
(10) Chen, C.; Zhang, X.; Wu, G.; Li, H.; Chen, H. Visible-light ultrasensitive solution-prepared layered organic-inorganic hybrid perovskite field-effect transistor. *Adv. Opt. Mater.* **2017**, *5* (2), 1600539.
(11) Zhang, F.; Zhang, H.; Zhu, L.; Qin, L.; Wang, Y.; Hu, Y.; Lou, Z.; Hou, Y.; Teng, F. Two-dimensional organic–inorganic hybrid perovskite field-effect transistors with polymers as bottom-gate dielectrics. *J. Mater. Chem. C* **2019**, *7* (14), 4004-4012.
(12) Zhu, H.; Liu, A.; Luque, H. L.; Sun, H.; Ji, D.; Noh, Y.-Y. Perovskite and conjugated polymer wrapped semiconducting carbon nanotube hybrid films for high-performance transistors and phototransistors. *ACS Nano* **2019**, *13* (4), 3971-3981.
(13) Gao, Y.; Wei, Z.; Yoo, P.; Shi, E.; Zeller, M.; Zhu, C.; Liao, P.; Dou, L. Highly stable

lead-free perovskite field-effect transistors incorporating linear π-conjugated organic ligands. *J. Am. Chem. Soc.* **2019**, *141* (39), 15577-15585.

(14) Zhu, H.; Liu, A.; Shim, K. I.; Hong, J.; Han, J. W.; Noh, Y. Y. High-performance and reliable lead-free layered-perovskite transistors. *Adv. Mater.* **2020**, *32* (31), 2002717.

(15) Zhu, H.; Liu, A.; Kim, H.; Hong, J.; Go, J.-Y.; Noh, Y.-Y. High-performance layered perovskite transistors and phototransistors by binary solvent engineering. *Chem. Mater.* **2020**, *33* (4), 1174-1181.

(16) Zhu, H.; Liu, A.; Zou, T.; Jung, H.; Heo, S.; Noh, Y.-Y. A Lewis base and boundary passivation bifunctional additive for high performance lead-free layered-perovskite transistors and phototransistors. *Mater. Today Energy* **2021**, *21*, 100722.

(17) Liang, A.; Gao, Y.; Asadpour, R.; Wei, Z.; Finkenauer, B. P.; Jin, L.; Yang, J.; Wang, K.; Chen, K.; Liao, P.; Zhu, C.; Huang, L.; Boudouris, B. W.; Alam, M. A.; Dou, L. Ligand-driven grain engineering of high mobility two-dimensional perovskite thin-film transistors. *J. Am. Chem. Soc.* **2021**, *143* (37), 15215-15223.

(18) Wei, Z.; Wang, K.; Zhao, W.; Gao, Y.; Hu, Q.; Chen, K.; Dou, L. A selenophene-containing conjugated organic ligand for two-dimensional halide perovskites. *Chem. Commun.* **2021**, *57* (87), 11469-11472.

(19) Shao, S.; Talsma, W.; Pitaro, M.; Dong, J.; Kahmann, S.; Rommens, A. J.; Portale, G.; Loi, M. A. Field-effect transistors based on formamidinium tin triiodide perovskite. *Adv. Funct. Mater.* **2021**, *31* (11), 2008478.

(20) Yang, W.; Park, G.; Liu, A.; Lee, H. B.; Kang, J. W.; Zhu, H.; Noh, Y. Y. Fluorinated organic A-cation enabling high-performance hysteresis-free 2D/3D hybrid tin perovskite transistors. *Adv. Funct. Mater.* **2023**, 2303309.

(21) Zhu, H.; Liu, A.; Shim, K. I.; Jung, H.; Zou, T.; Reo, Y.; Kim, H.; Han, J. W.; Chen, Y.; Chu, H. Y. High-performance hysteresis-free perovskite transistors through anion engineering. *Nat. Commun.* **2022**, *13* (1), 1741.
